# Estimating the Intra-taxa Diversity, Population Genetic Structure, and Evolutionary Pathways of *Cryptococcus neoformans* and *Cryptococcus gattii*

**DOI:** 10.3389/fgene.2018.00148

**Published:** 2018-04-24

**Authors:** Marina Muñoz, Milena Camargo, Juan D. Ramírez

**Affiliations:** ^1^Grupo de Investigaciones Microbiológicas-UR (GIMUR), Programa de Biología, Facultad de Ciencias Naturales y Matemáticas, Universidad del Rosario, Bogotá, Colombia; ^2^Centro de Tecnología en Salud (CETESA), Upqua SAS, Bogotá, Colombia; ^3^Posgrado Interfacultades Doctorado en Biotecnología, Facultad de Ciencias, Universidad Nacional de Colombia, Bogotá, Colombia; ^4^Departamento de Biología Molecular e Inmunología, Fundación Instituto de Inmunología de Colombia, Bogotá, Colombia; ^5^Doctorado en Ciencias Biomédicas y Biológicas, Universidad del Rosario, Bogotá, Colombia

**Keywords:** *C. neoformans*, *C. gattii*, multilocus sequence typing (MLST), allelic diversity, population structure, evolutionary history

## Abstract

Members of the *Cryptococcus* complex, includes *Cryptococcus neoformans* (most common fungal infection of the brain) and *Cryptococcus gattii* (high-impact emerging pathogen worldwide). Currently, the fungal multilocus sequence typing database (Fungal MLST Database) constitutes a valuable data repository of the genes used for molecular typing of these pathogens. We analyzed the data available in the Fungal MLST Database for seven housekeeping genes, with the aim to evaluate its contribution in the description of intra-taxa diversity, population genetic structure, and evolutionary patterns. Although the Fungal MLST Database has a greater number of reports for *C. neoformans* (*n* = 487) than for *C. gattii* (*n* = 344), similar results were obtained for both species in terms of allelic diversity. Phylogenetic reconstructions revealed grouping by molecular type in both species and allowed us to propose differences in evolutionary patterns (gradualism in the case of *C. neoformans* and punctuated evolution in the case of *C. gattii*). In addition, *C. neoformans* showed a population genetic structure consisting of 37 clonal complexes (CCs; CC1 being predominant), high crosslinking [without sequence type (ST) grouping by molecular type], marked divergence events in phylogenetic analysis, and few introgression events (mainly between VNI and VNIV). By contrast, *C. gattii* showed 50 CCs (with greater homogeneity in ST number by CC) and clustering by molecular type with marked crosslinking events in phylogenetic networks being less evident. Understanding relationships at the molecular level for species of the *Cryptococcus* complex, based on the sequences of the housekeeping genes, provides information for describing the evolutionary history of these emerging pathogens.

## Introduction

The public health importance of the *Cryptococcus* complex has been confirmed by epidemiological data, indicating more than 620,000 deaths annually (Springer et al., 2014). In particular, in HIV-infected individuals, the mortality rate associated with *Cryptococcus* is high (181,100 deaths annually) and the main cause of meningitis (Lin, 2009; Rajasingham et al., 2017). The clinical response is hampered by the limited treatment options and an aggressive infectious process that responds slowly to treatment, meaning that long periods of drug administration are required, potentially resulting in the emergence of resistance to the available antifungal drugs (Lin, 2009; Espinel-Ingroff and Kidd, 2015). At the diagnostic level, the detection of infections by species of *Cryptococcus* allows for the timely treatment of opportunistic infections. At present, the implementation of molecular tests within pathogen identification schemes has contributed to our understanding of population structure at the epidemiological level, as well as providing insight into the follow-up of persistent infections or relapses (Abassi et al., 2015; Perfect and Bicanic, 2015).

The *Cryptococcus* complex is composed of *Cryptococcus neoformans* and *Cryptococcus gattii*, currently recognized as distinct species (Kwon-Chung and Varma, 2006; Byrnes et al., 2011). Molecular studies have estimated that these species diverged approximately 37.5 million years ago and share an identity of 87%, which defines them as separate species (Lin, 2009; Byrnes et al., 2011; D’Souza et al., 2011). *C. neoformans* is recognized as the most widely distributed species worldwide, mainly infecting immunocompromised individuals (Barreto de Oliveira et al., 2004; Idnurm et al., 2005). This pathogen has been the focus of many studies aimed at identifying the mechanisms related to pathogenesis, with the goal of identifying potential therapeutic targets (Lin, 2009; Ding et al., 2016; Taylor-Smith and May, 2016). However, *C. gattii* has also emerged as a pathogen capable of affecting immunocompetent individuals, which has prompted public health concern (Perfect et al., 2010; Espinel-Ingroff and Kidd, 2015). Initially regarded as being restricted to tropical and subtropical regions, this pathogen was subsequently found to be associated with outbreaks in temperate regions, involving infections not associated with immunosuppression (Stephen et al., 2002; Datta et al., 2009; Ngamskulrungroj et al., 2011). These reports have focused attention on the virulence of *C. gattii* as a primary and emerging pathogen, with wide geographical distribution and an annual increase in the number of cases (Kidd et al., 2004; Byrnes et al., 2010, 2011).

**FIGURE 1:**
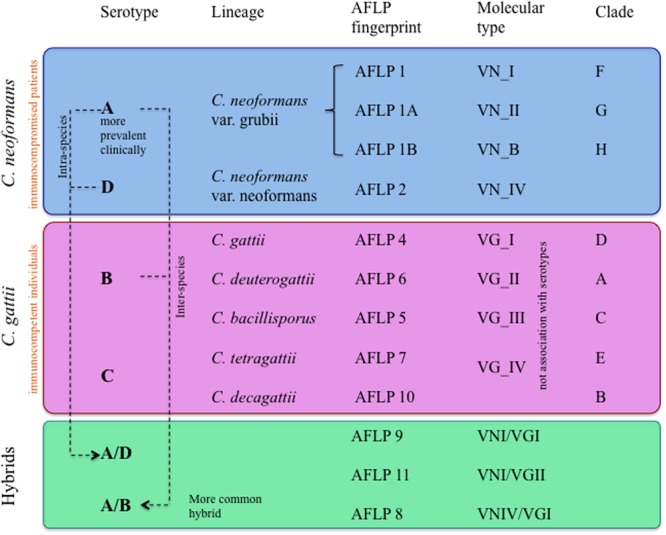
Classification of the variants, serotypes, genotypes, and hybrids of *Cryptococcus neoformans* and *Cryptococcus gattii.*

Initially, *Cryptococcus* species complex was classified serologically in four serotypes (A–D) (Espinel-Ingroff and Kidd, 2015). Subsequently, the complex was divided into serotypes and latter into lineages by means of the Amplified Fragment Length Polymorphism (AFLP) fingerprint (Bovers et al., 2008a; Chen et al., 2014). At the level of molecular types, *C. neoformans* members have been grouped into four variants: VNI, VNII, and VNB, which includes serotype A (comprising *var. grubii* lineages), and VNIV of serotype D (*var. neoformans*). In the case of *C. gattii*, four variants exist, VGI and II, including serotype B (*var. gattii* and *var. deuterogattii*, respectively), and variants VGIII and VGIV of serotype C (*var. bacillisporus* and *var. tetragattii*). Hybrids between *C. neoformans* and *C. gattii* (for example, VGI–VNI and VGII–VNIV) have been reported, which have contributed to the genetic variability in the genus *Cryptococcus* (Byrnes et al., 2011; Farrer et al., 2015; Ding et al., 2016). A summary of the current classification of *Cryptococcus* derived from various experimental approaches is described in **Figure 1**.

Regarding the *Cryptococcus* complex distribution, *C. neoformans* var. *grubii* (VNI-II and VNB), considered the causative agent of cryptococcosis, is more prevalent and widely distributed worldwide compared with *C. neoformans var. neoformans* (VNIV), which has been predominantly isolated in Europe (Desnos-Ollivier et al., 2015; Cogliati et al., 2016). Among the members of *C. gattii*, the most widely distributed are *var. gattii* (VGI) and *C. gattii var. deuterogattii* (VGII), associated mainly with outbreaks in the Northwest region of North America among immunocompetent individuals, whereas *var. tetragattii* (VGIV) appears to be the least common and is associated with outbreaks in Africa among immunocompromised individuals (Chen et al., 2014).

During the last two decades, multilocus sequence typing (MLST) tools have been implemented for the genotyping of pathogenic microorganisms, with the aim of describing their molecular epidemiology and monitoring outbreaks (MacCannell, 2013). This approach describes the allelic variation at multiple housekeeping loci, through independent amplification, followed by direct sanger sequencing, which together allow a sequence type (ST) to be assigned for each genotype (Cooper and Feil, 2004). Although this tool was initially described in bacteria, it has been positioned as a highly accurate and discriminative approach, even in eukaryotic organisms and species for which classification is difficult (prokaryotes and eukaryotes) (Almeida and Araujo, 2013). The scheme currently accepted for genotyping *Cryptococcus* species was proposed by Meyer et al. (2009) and is performed based on the consensus of the International Society of Human and Animal Mycoses (ISHAM) (Meyer et al., 2009). This MLST scheme analyzes the genes encoding the following proteins: *CAP59* (capsular-associated protein), *GPD1* (glyceraldehyde-3-phosphate dehydrogenase), *LAC1* (laccase), *PLB1* (phospholipase), *SOD1* (Cu, Zn superoxide dismutase), and *URA5* (orotidine monophosphate pyrophosphorylase), in addition, the *IGS1* (ribosomal RNA intergenic spacer) region is included (Meyer et al., 2009).

Although the MLST data from fungal pathogens is recent (i.e., *Candida* species and *Aspergillus fumigatus*), different efforts have developed and implemented a standardized MLST scheme for the rapid genotyping of *Cryptococcus* complex, through appropriate allele type (AT) identification and ST number assignment. The International Fungal Multilocus Sequence Typing Database ‘Fungal MLST Database’^1^ currently represents the most complete repository of MLST data for fungi, where publicly deposited data of *Cryptococcus* species is available. In light of the severe clinical manifestations of cryptococcosis, the wide genetic diversity previously reported for *Cryptococcus* species and the lack of a consensus for intra-species classification. This study used sequences of seven housekeeping genes employed in the *Cryptococcus* species’ MLST (publicly available in the Fungal MLST Database), with the aim of determining phylogenetic relationships, revealing the population genetic structure, and inferring the signatures of evolutionary patterns at the intra-taxa level for these species (in terms of plausible recombination).

## Materials and Methods

### Data Download

Three sets of data were organized from the information publicly available in the Fungal MLST Database (last accessed: 09-09-2017). The first set corresponded to the allelic profiles of the total STs reported, which were identified through the available searching tool for each species: in the case of *C. neoformans* http://mlst.mycologylab.org/Biolomics.aspx?Table=Sequence%20types%20C.%20neoformans and in the case of *C. gattii* http://mlst.mycologylab.org/Biolomics.aspx?Table=Sequence%20types%20C.%20gattii. The allelic combinations were used to construct an excel file with the MLST profiles for each species.

The second set of data was composed of the sequences of the alleles reported for each gene, which were identified from the MLST profiles file and then manually downloaded using the Fungal MLST Database search tool. Allele sequences were used to construct an allele sequences file for each housekeeping gene in each species. Finally, a third set of data corresponded to the sequences of the total STs reported, which were manually downloaded following the links available in the search tool, which was then used to construct a fasta file with the concatenated sequence for each ST. The fasta files were tagged with the molecular type information assigned in the Fungal MLST Database and later included in the ST sequences (concatenated and per gene) for each of the species.

All of the sequences retrieved were verified by a second investigator, to eliminate any errors generated during the manual unloading process. The files with sequences (ST sequences) were aligned using the PyNAST method, a python implementation of the NAST alignment algorithm (Caporaso et al., 2010), selected for this set of data to favor the best-match in a pre-aligned database of sequences aligning each provided sequence as a “candidate” sequence. The requirements to match template sequences within PyNAST method were a percentage of identity of 75% and a minimum sequence length of 150 bp.

### Determining the Allelic Diversity

As a first step in the analysis of the MLST data for the species in the *Cryptococcus* complex, descriptive statistics were used to evaluate the percentage of STs belonging to each molecular type among the total STs reported and the number of alleles reported for both species and molecular type. Second, we conducted analysis aimed at evaluating the allelic diversity of the six core metabolic genes and one non-housekeeping gene by calculating genetic diversity indexes as an indicator of the selection pressure to which they are subjected (Cooper and Feil, 2004). Hence, population genetic parameters were determined for each gene used in the MLST scheme, including the total number of mutations (Eta), the number of haplotypes (h), haplotype diversity (Hd), nucleotide diversity (π), Theta (per site) (k) as calculated using the DnaSP software (v5), and the index of association (IA) as calculated using the LInkage ANalysis software (v3.7). Third, the utility of the MLST scheme for each species was determined by calculating the typing efficiency – TE (number of STs identified by polymorphic site) and discrimination power – DP (number of STs identified by total number of isolates), with a 95% confidence interval (CI) using the MLSTest software version 1.0.1.23. These evaluate the performance of the markers that make up an MLST scheme. A comparison of TE and DP results was carried out between species to identify statistically significant differences (Fisher test). These parameters allow to define the number of genotypes by loci present and determine the probability that they are closely related, allowing an adequate classification (Trindade et al., 2003; Tomasini et al., 2013; Varni et al., 2014). Additionally, a scheme optimization analysis was developed to identify the optimum number of loci and the best combination required to find the highest number of STs.

### Phylogenetic Analysis

To identify signatures of the evolutionary history (species emergence) of the evaluated species, phylogenetic inferences were made from the files with the concatenated sequence of the seven housekeeping genes for all STs reported for each species. *Filobasidiella depauperata* strain CBS7855 was selected as an outgroup (Accession Number: GU131349.1) and considered a closely related species predominantly used in previous studies (Ngamskulrungroj et al., 2009); a database search of the homologous sequences for the seven markers was carried out by organisms using the “Choosing search set” tool, from the National Center for Biotechnology Information. The identified sequences were verified through genus-specific search from RefSeq (Pruitt et al., 2012). The concatenated sequences of the homologous genes in *F. depauperata* strain were included in the analysis. The complete set of sequences for each species was aligned using multiple sequence comparison by log-expectation (MUSCLE) (Edgar, 2004). A maximum-likelihood tree based on this alignment was constructed using FastTree 2.1 (Price et al., 2009), considering Jukes–Cantor as the model of nucleotide evolution. The robustness of the nodes was evaluated using the bootstrap (BT) method with 1,000 replicates. A cluster was defined from the nodes with BT results of >95%, which included the majority of members previously defined as having the same molecular type. The graph visualization of the phylogenetic trees was conducted using the web-based tool Interactive Tree Of Life V3^2^ (Letunic and Bork, 2016).

### Multilocus Sequence Analysis (MLSA)

Analysis based on MLST sequences was developed with the purpose of inferring the amount and distribution of genetic variation within and among populations, as an indicator of the genetic population structure (McDonald, 1997). Hence, grouping tools were used to establish the ancestral molecular type, clonal complexes (CCs), singletons present, and the relationship between established groups; a preliminary step in describing the population structure of *Cryptococcus* complex members was applied with the eBURST V3 package^3^, using as the input the allelic profiles for each species. BURST is directed to the analysis of closely related sequences connected by changes in unique nucleotides, and therefore establish relationship with other STs in terms of single locus variants (SLVs), double locus variants (DLVs), and triple locus variants (TLVs). Additionally, this approach allows CCs to be assigned as representative of bound and closely related clusters, which also allows ST founders to be established for each CC, along with them. Minimal expansion trees were also generated using the geoBURST algorithm (Francisco et al., 2009), as a verification strategy of the definition of CCs and their organization, and also as an evolutionary model represented graphically.

### Recombination Analysis

A panel of analyzes aimed at evaluating the effect of the recombination of the seven housekeeping genes used for the MLST scheme was developed. Firstly, phylogenetic networks using the Neighbor-Net method (Huson and Bryant, 2006), available in the SplitsTree4 package (Version 4.14-4) with 1000 iterations, were developed with the purpose of identifying the molecular rearrangements among the sequences, represented by crosslinking events. Finally, inferences about the population structure of the *Cryptococcus* complex species were reported from the allelic profiles of the MLST scheme using the STRUCTURE estimation method (Rieux et al., 2011), which was implemented using an admixture model of ancestry with a length of burning period with 60,000 iterations and considering the Markov chain Monte Carlo (MCMC) algorithm with a number of reps after burning of 600,000. Each molecular type was considered as a cluster *a priori* (K: 4). Additionally, analyzes in the Recombination Detection Program version 4 (RDP4) were developed (Martin et al., 2015) to detect and characterize recombination signals from the complete set of concatenated sequences for each species. RDP4 includes the following methods: RDP, GENECONV, BootScan, Maximum Chi Square (MaxiChi), Chimaera, Sister Scanning (SiScan), 3Seq, VisRD method, and BURT. A recombination breakpoint matrix plot was generated for each species and the description of different recombination events was conducted, in terms of recombination scores (for recombinants and their parents: major and minor). Recombination signals attributable to a process other than recombination were identified in the events that corresponded.

## Results

### Description of the Data Reported in the MLST-db

**FIGURE 2:**
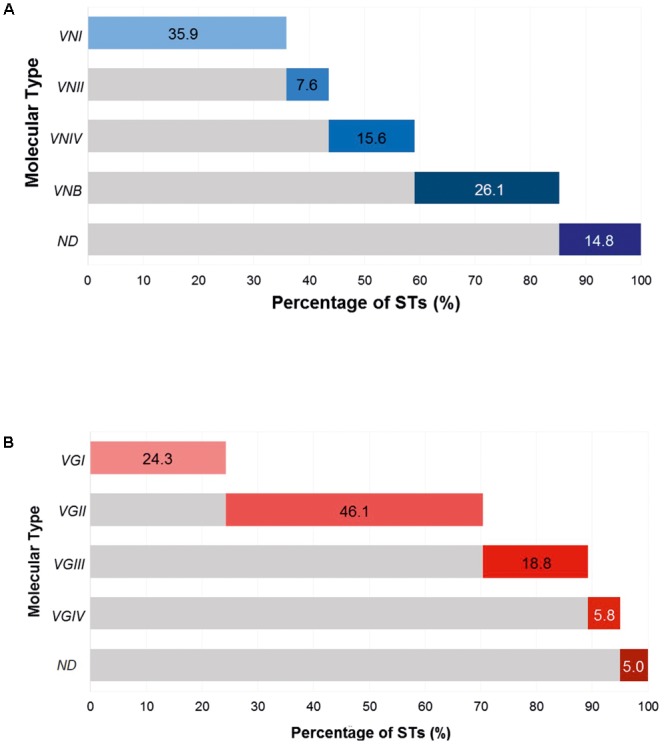
Cumulative frequency of molecular types of the *Cryptococcus* complex. **(A)** *Cryptococcus neoformans*. **(B)** *Cryptococcus gattii.*

A total of 849 STs of the *Cryptococcus* complex were identified in the Fungal MLST Database on the last access date, corresponding to 487 STs for *C. neoformans*, higher than those found for *C. gattii* (362 STs). In the case of *C. neoformans*, 175 STs were classified as VNI (35.9%; 95% CI: 5.4–10.3), followed by VNB with 127 STs (26.7%, 95% CI: 22.2–30.2); in addition, 72 STs (14.8%, 95% CI: 11.7–18.2) were not assigned (NA) to any molecular type in the Fungal MLST Database (not determined, ND); distributions of the remaining molecular types are shown in **Figure 2A**. In the case of *C. gattii*, the STs reported were more frequently assigned to molecular type VGII, which has 167 STs (46.1%; 95% CI: 40.9–51.4), while the lowest reported frequency was for molecular type VGIV with 21 STs (5.8%, 95% CI: 3.6–8.7) and 18 STs (5.0%, 95% CI: 2.9–7.7) could not be assigned to a molecular type (ND), percentages of the other molecular types are described in **Figure 2B**. From this last group, 16 STs did not have any information on their allelic profile in the Fungal MLST Database and were excluded from the analyzes. Therefore, the data set for *C. gattii* consisted of 344 STs.

**FIGURE 3:**
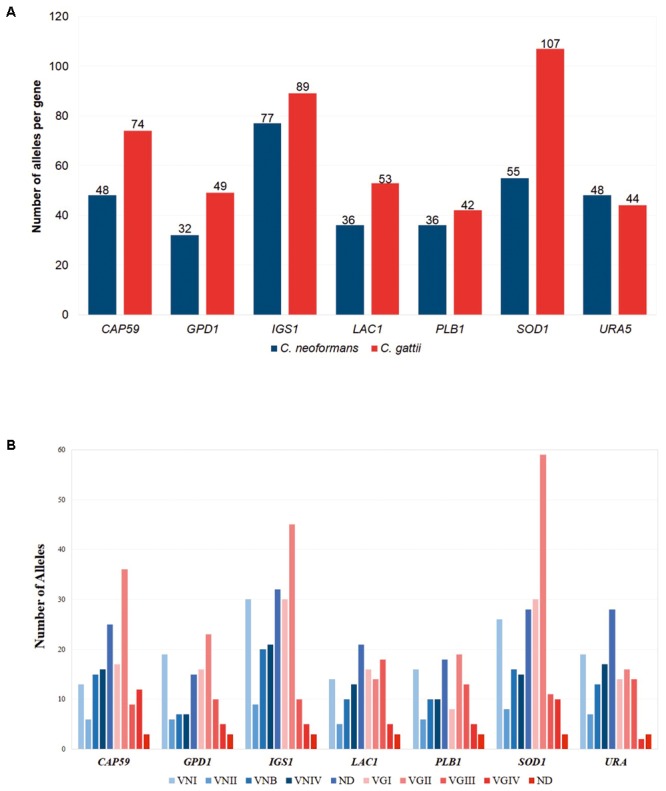
Comparison of the number of alleles found for each of the MLST markers. **(A)** According to the species, *C. neoformans* and *C. gattii*. **(B)** According to the molecular type.

As for the number of alleles identified for MLST genes used in the scheme, we found that *C. gattii* possessed more alleles for all of the genes, except for *URA5* where the number of alleles was similar between the two species. The highest number of alleles were detected for the *SOD1* and *IGS1* genes in the two species, and these molecular markers were considered as indirect indicators of diversity. The results indicated that despite using the same scheme for the classification of these organisms by MLST, a number of major gene alleles were observed for *C. gattii* compared with *C. neoformans*. These results are shown in **Figure 3A**. Additional analysis to identify the number of alleles by molecular type was conducted and showed that the number of alleles was consistent across all genes in *C. neoformans*, with a slight increase in the ST group defined as ND, whereas for *C. gattii* marked peaks were observed for the VGII allele relating to the most frequent molecular type. The detailed results of this analysis are described in **Figure 3B**.

### Genetic Diversity and Utility of MLST Scheme Markers

Multiple alignments of the concatenated sequences (>4200 bp in length) of the seven markers for the *Cryptococcus* complex species were analyzed. For *C. neoformans*, of the 487 sequences analyzed, the results showed the presence of 659 segregating (polymorphic) sites (S) with an haplotype diversity (Hd) of 0.999; *CAP59* and *IGS1* had the lowest percentages of identity (<32%). However, these loci had higher values for the remaining indexes, with the highest polymorphism markers identifying 47 haplotypes each (Hd: 1,000 and 0.975, respectively) for Eta 367 and 496, respectively, and an average number of nucleotide differences (*k*) of 43,620 and 54,803, respectively. By contrast, the *GPD1* and *PLB*1 markers with identity percentages >82% showed the lowest values when identifying only 30 haplotypes (Hd: 0.996 and 0.987, respectively) for Eta of 78 and 63, respectively, and *k*-values of 19,220 and 15,600 (**Table 1**). The indexes for *C. gattii* calculated for 344 sequences showed the presence of 642 segregating (polymorphic) sites and a Hd of 0.999; and the markers for this species showed higher values in terms of identity, number of haplotypes, and haplotypic diversity to those observed for *C. neoformans*. *SOD1* and *CAP59* were associated with greater identification of haplotypes (101 and 66, respectively) and haplotypic diversity (Hd: 0.998 and 0.997, respectively), whereas the *URA*5 marker was associated with low levels of haplotype identification (39, Hd: 0.995). Furthermore, the Eta and *k URA5* indexes showed higher values of 427 and 31,886, respectively, whereas *CAP59* presented the lowest values for these indexes (Eta: 90 and *k*: 16,775) (**Table 1**). In addition, diversity indexes showed that for *C. neoformans*, molecular type VNI presented the highest number of haplotypes (137) and the lowest nucleotide diversity (0.002); whereas for *C. gatti*, VGII presented the highest number of haplotypes (147), while the lowest diversity was presented in VGIV (Supplementary Table S1).

**Table 1:** Diversity indexes for the markers in the MLST schemes for *Cryptococcus* species.

	*Cryptococcus neoformans*								*Cryptococcus gattii*							
	*CAP59*	*GPD1*	*IGS1*	*LAC1*	*PLB1*	*SOD1*	*URA5*	All loci	*CAP59*	*GPD1*	*IGS1*	*LAC1*	*PLB1*	*SOD1*	*URA5*	All loci
Sequence length	655	555	799	478	534	545	661	4230	558	551	767	480	713	717	718	4254
Number of sequences used	48	32	77	36	36	55	48	487	74	49	89	53	42	107	44	344
Identity (%)	32.06	83.06	14.23	82.85	84.83	76.51	32.52	–	85.66	81.85	58.15	79.17	26.99	78.38	32.87	–
Total number of sites (excluding sites with gaps/missing data)	543	535	485	467	515	509	521	3775	556	537	573	466	528	687	630	3938
Number of polymorphic (segregating) sites (S)	333	74	379	71	62	94	311	659	78	86	124	86	339	125	394	642
Haplotypes	47	30	47	33	30	43	45	424	66	42	63	43	40	101	39	315
Haplotype diversity (Hd)	1.000	0.996	0.975	0.995	0.987	0.991	0.997	0.999	0.997	0.986	0.975	0.988	0.998	0.998	0.995	0.999
Nucleotide diversity (Pi)	0.080	0.035	0.113	0.048	0.030	0.053	0.051	0.030	0.030	0.040	0.035	0.042	0.059	0.028	0.050	0.030
Number of mutations (Eta)	367	78	496	74	63	101	340	700	90	98	137	90	361	133	427	696
Theta (per site) from Eta	0.153	0.034	0.208	0.038	0.029	0.043	0.147	0.027	0.033	0.040	0.047	0.042	0.158	0.036	0.155	0.027
Average number of nucleotide differences (*k*)	43.620	19.220	54.803	22.551	15.600	27.145	27.023	115.861	16.775	21.888	20.498	20.033	31.262	19.261	31.886	120.702
Index of association (IA)	0.2292								0.2176							

Using MLSTest software, parameters were calculated to establish the utility of the markers used in the MLST including the number of polymorphisms, the TE, and the DP. The results showed higher TE levels for all markers of *C. gattii*, with *CAP59* considered as the most efficient (0.926). The DP levels were higher for most markers of *C. gattii*, with *IGS1* considered as higher in *C. neoformans* and *C. gattii* (0.917 and 0.974, respectively) (**Table 2A**). The comparisons of TE and DP between species were statistically significant. Scheme optimization analysis showed that the seven genes used represent the optimum number of loci required to identify the largest number of STs. The results of the *in silico* analysis showed the discrimination of 487 STs in the case of *C. neoformans* and 344 STs for *C. gattii* (**Table 2B**), representing 100 and 95.0% of the STs, with respect to the total of the STs reported at the date of conducting the analysis for each species, respectively.

**Table 2:** Analysis of the markers used within the MLST scheme for species of the *Cryptococcus* complex.

(A) Calculating the typing efficiency and discriminatory power									
	Species	*CAP59*	*GPG1*	*IGS1*	*LAC1*	*PLB1*	*SOD1*	*URA5*	STs
Number of alleles	*C. neoformans*	48	32	77	36	36	55	48	487
	*C. gattii*	74	49	89	53	42	107	44	344
Number of polymorphisms	*C. neoformans*	61	94	562	83	83	131	100	1114
	*C. gattii*	81	100	359	99	83	156	80	958
Typing efficiency^∗^	*C. neoformans*	0.77	0.34	0.176	0.434	0.458	0.443	0.5	0.323
	*C. gattii*	0.926	0.49	0.253	0.545	0.506	0.686	0.55	0.482
Discriminatory power^∗^ (95% CI)	*C. neoformans*	0.881	0.878	0.917	0.907	0.911	0.83	0.91	1
		(0.861–0.901)	(0.864–0.892)	(0.898–0.935)	(0.893–0.92)	(0.903–0.92)	(0.8–0.859)	(0.894–0.926)	1(1–1)
	*C. gattii*	0.94	0.906	0.974	0.873	0.931	0.969	0.901	1
		(0.929–0.951)	(0.888–0.925)	(0.969–0.979)	(0.843–0.902)	(0.92–0.941)	(0.961–0.977)	(0.88–0.92)	1(1–1)
**(B) Analysis of scheme optimization and the optimum number of loci**									
**Number of loci**	**Number of combinations**	**Loci**				**Minimum number of STs found**	**Mean number of STs found**	**Maximum number of STs found**

		***C. neoformans***						
1	7	*IGS1*				32	51	99
2	21	*IGS1, SOD1*				99	148	207
3	35	*IGS1, LAC1, SOD1 IGS1, SOD1, URA5*				187	244	293
4	35	*IGS1, LAC1, PLB1, SOD1 IGS1, LAC1, PLB1, URA5*				280	326	363
5	21	*IGS1, LAC1, PLB1, SOD1, URA5*				354	394	425
6	7	*GPD1, IGS1, LAC1, PLB1, SOD1, URA5*				424	447	462
7	1	*CAP59, GPD1, IGS1, LAC1, PLB1, SOD1, URA5*				487	487	487

		***C. gattii***						
1	7	*SOD1*				42	66	107
2	21	*IGS1, SOD1*				97	145	208
3	35	*IGS1, LAC1, SOD1*				155	213	256
4	35	*IGS1, LAC1, PLB1, SOD1*				213	263	289
5	21	*CAP59, IGS1, LAC1, PLB1, SOD1*				269	298	313
6	7	*CAP59, IGS1, LAC1, PLB1, SOD1, URA5*				310	323	330
7	1	*CAP59, GPD1, IGS1, LAC1, PLB1, SOD1, URA5*				344	344	344

*^∗^These results showed a statistically significant difference between both species.*

### Phylogenetic Analysis

**FIGURE 4:**
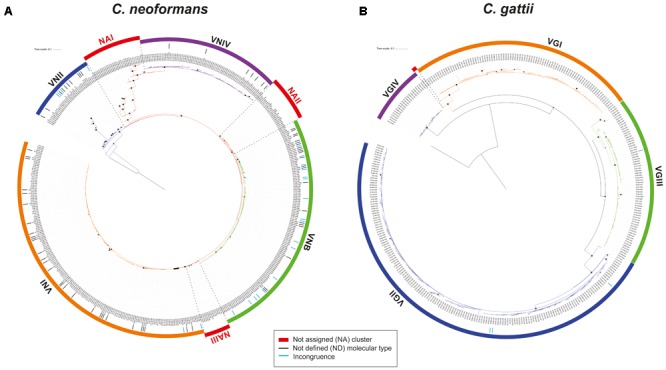
Phylogenetic analysis of the molecular type for **(A)** *C. neoformans* and **(B)** *C. gattii*. Trees were constructed using the maximum-likelihood method and Jukes–Cantor as the model of nucleotide evolution. The black points represent bootstrap values of >95% and the size of the points is proportional to the BT (with increments of 1 px per BT unit). The clusters were delimited considering the nodes.

The phylogenetic reconstructions obtained from the concatenated sequence of the seven housekeeping genes used for the MLST scheme allowed us to identify four independent clusters for *C. neoformans* broadly corresponding to the four molecular types assigned to each ST (**Figure 4A**). However, 72 STs could not be assigned to any of these clusters, and it was not possible to assign these STs to a molecular type. Hence, they were defined as “NA.” These NA STs were located in three separate areas of the phylogenetic tree and were assigned to three different groups (designated 1–3). Based on the groupings obtained in this phylogeny, a molecular type was assigned to 69 of the 107 STs that did not have this information in the Fungal MLST Database (equivalent to 64.5% of this group). In addition, molecular type information was compared for other STs, which showed a total of 58 incongruities (where the assigned molecular type does not match the clade to which it belongs in the phylogenetic reconstruction), equivalent to 16.8% of the total STs with an assigned molecular type (*n* = 346).

In the case of *C. gattii*, the results of the phylogenetic reconstruction (**Figure 4B**) were more in agreement with the information on molecular type available in the Fungal MLST Database, which identified the four clusters and detected only four incongruences in the classification equivalent to 1.2% of the total STs with information on molecular type (*n* = 342). The two STs defined as ND with information from the allelic profile were the only STs that could not be assigned to a molecular type.

### Multilocus Sequence Analysis and the Population Structure

**FIGURE 5:**
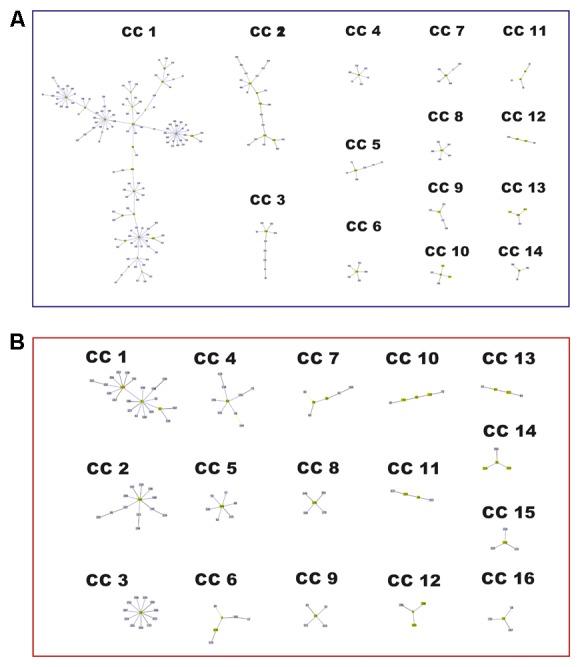
Network structure observed in a BURST diagram for the species of the *Cryptococcus* complex. **(A)** *Cryptococcus neoformans*. **(B)** *Cryptococcus gattii.*

The implementation of the eBURST algorithm from the allelic profiles reported for each species in the Fungal MLST Database identified 37 CCs among the 281 STs of *C. neoformans*, of which 14 included 4 or more STs (**Figure 5A**), while the remaining 206 STs were designated as singletons. CC1 included 138 STs, assigned predominantly to molecular type VNI (111 STs, equivalent to 80.4%). CC2 included a total of 23 STs, represented mainly by VNB (19 STs, corresponding to 79.3%). The remaining CCs included ≤10 STs (**Figure 5A** and Supplementary Table S2). In the case of *C. gattii*, the results of the algorithm eBURST analysis showed that 191 STs were grouped into 50 CCs, of which 16 included 4 or more STs (**Figure 5B**) and 153 STs corresponded to singletons. CC1 included the largest number of STs (22 STs), corresponding to VGI, followed by CC2 with 13 STs (all VGI), and CC3 with 12 STs (mostly VGII), the remaining complexes included ≤9 STs (**Figure 5B** and Supplementary Table S3).

### Recombination Signals Analyzes

**FIGURE 6:**
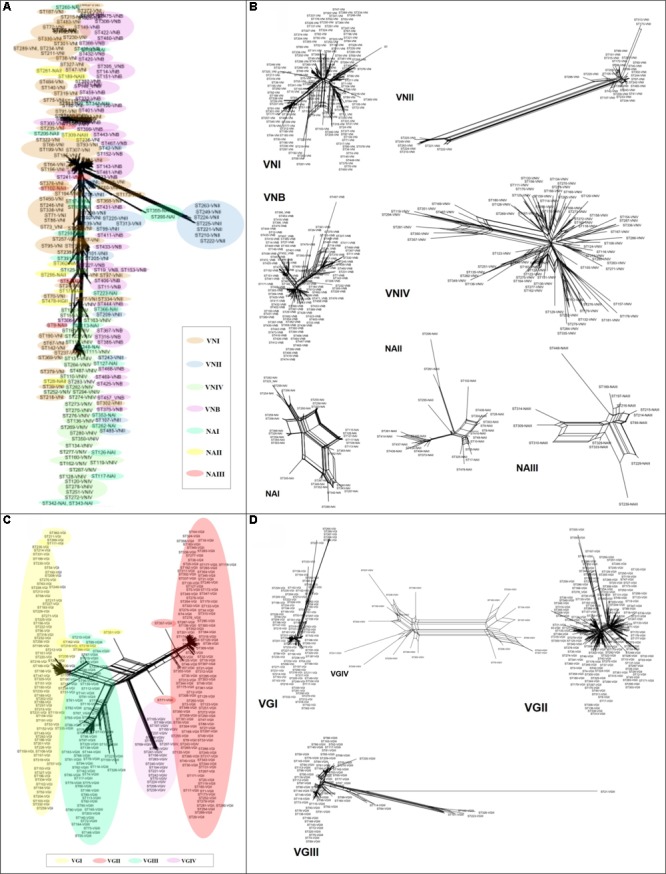
Phylogenetic networks based on the neighbor-net algorithm of the concatenated sequences. **(A)** *Cryptococcus neoformans.* **(B)** Molecular types of *C. neoformans*. **(C)** *Cryptococcus gattii.* **(D)** Molecular types of *C. gattii.*

Alignments with concatenated sequences were used to reconstruct phylogenetic networks based on the neighbor-net algorithm to evaluate the existence of plausible events of recombination. The analysis was conducted for both STs and molecular types for each of the species. The results revealed that the phylogenetic network of *C. neoformans* displayed a high number of crosslinking events, but interestingly did not show the clustering of STs by molecular type/cluster (**Figure 6A**). At the marker level, high divergence was detected within this species, without reticulation events (Supplementary Figure S1A). The highest levels of crosslinking were observed for molecular type VNIV, followed by VNI. There is a highly divergent ST group within molecular type VNII, but greater crosslinking was identified when analyzing the NA groups, which was not possible to assign to any of the major molecular types/clusters (**Figure 6B**).

**FIGURE 7:**
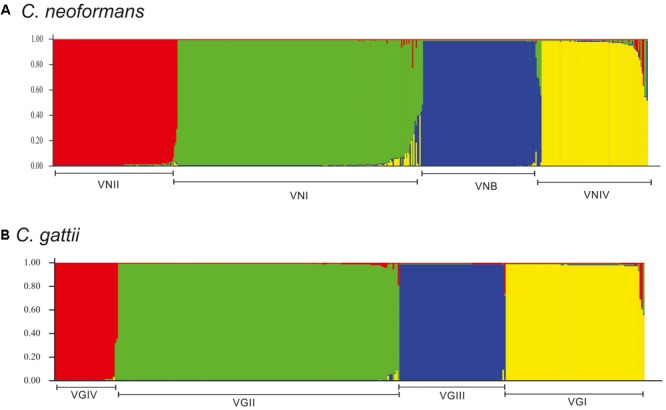
Structure analysis for *k* = 4 for the molecular types of each of the species of the *Cryptococcus* complex. **(A)** *C. neoformans* and **(B)** *C. gattii*. The bottom line in each plot represents the population corresponding to each molecular type (where each name has been assigned).

In the case of *C. gattii*, crosslinking was observed toward the center of the network, but with a more defined pattern of grouping by molecular type (**Figure 6C**). Most of the markers (*CAP59, GPD1, IGS1, LAC1*, and *SOD1*) for this species exhibited marked crosslinking events (Supplementary Figure S1B). Analysis at the molecular type level showed that VGIV exhibited marked crosslinking events, whereas VGIII had some members displaying a high degree of divergence (**Figure 6D**). Finally, the estimated log of probability for the data analyzed with STRUCTURE for *k* = 4 (using an admixture model) showed that *C. neoformans* had undergone introgression events, mainly between VNI and VNIV (**Figure 7A**). In the case of *C. gattii*, although the introgression events were less evident, it is possible to detect cases of plausible introgression between VGI and VGII (**Figure 7B**). These findings agree with previous reports that identified introgression events between VGI and VGII (Billmyre et al., 2014). However, it is not possible to distinguish between introgression and the emergence of hybrids, because the genetic information available is limited. As a next step, analyzes in RDP program were developed to identify possible recombination events and to characterize the potential source of them. The number of recombination events found in *C. neoformans* was twice the ones found in *C. gattii* (27 and 12 events, respectively) (Supplementary Figure S2). The characterization of these recombination events allowed to identify 20 potential recombinants in *C. neoformans*, a high number of signals attributable to a process other than recombination and different misidentification of breakpoints, which could represent evidence associated with the existence of hybrids (Supplementary Table S4). In the case of *C. gattii*, although signals attributable to a process other than recombination were identified, only one possible recombinant was detected (ST-100-VGIII; Supplementary Table S5).

## Discussion

Since the two main species of the *Cryptococcus* complex have a significant impact on public health, a large number of investigations have been undertaken to assess features such as the geographical and temporal distribution of these organisms, intra-specific level variations, and infections that affect dissimilar target populations (O’Meara and Alspaugh, 2012; Qiu et al., 2012; Almeida et al., 2015). Currently, a set of reference genomes for *Cryptococcus* complex species are available (Inglis et al., 2014) and genomics epidemiology studies have been developed (Vanhove et al., 2017). However, the strategy of MLST-based typing (followed by its corresponding analyzes) presented the following advantages: (i) it continues to be easy to access, especially in low-income countries where the whole-genome sequencing (WGS) is still restricted; (ii) requires basic bioinformatic efforts, compared with the WGS analysis, a component that still represents a challenge; (iii) there are curated databases where the findings obtained from isolates of different origins are centralized; and (iv) it presents high discriminatory power and efficiency in the typing between the different molecular types of *C. gattii* and *C. neoformans*.

In this context, the current study used information in MLST databases as a tool to analyze the intra-taxa diversity, population genetic structure, and evolutionary pathways of *Cryptococcus*. The data obtained from this study confirm the power of discrimination of the seven molecular markers included in *Cryptococcus* sp. MLST scheme. However, studies that support the results obtained here, as well as allowing a better understanding of the events of recombination, diversity, adaptation to new niches, and fine-scale phylogenetic analysis, should be carried out (Gillece et al., 2011; Engelthaler et al., 2014; Farrer et al., 2015; Desjardins et al., 2017; Rhodes et al., 2017).

In general, the findings of this analysis showed that in the case of *C. neoformans*, the molecular type most frequently reported in the database was VNI, followed by VNB (**Figure 2B**). Cryptococcosis resulting from infection with the molecular type of serotype A (mainly VNI) has been consistently reported as having the highest prevalence and distribution worldwide (Cogliati, 2013). In the case of *C. gattii*, the molecular types VGII and VGI proved to be the most frequent molecular types causing infection (**Figure 2B**), which agreed with data previously reported in the literature (Cogliati, 2013; Chen et al., 2014). These lineages have been associated with a higher impact globally (34 and 47%, respectively), with outbreaks in the northwest of North America, Australia (northern region), and Papua New Guinea, mainly affecting immunocompetent hosts and aboriginal populations in Australia (Byrnes et al., 2011; Chen et al., 2014). The VGIII and VGIV genotypes are associated with infections in immunocompromised patients, with frequencies of occurrence of 11 and 8%, respectively. These genotypes have been linked to outbreaks in sub-Saharan Africa and the United States; additionally, these genetic groups have shown similar epidemiological profiles to *C. neoformans* (Byrnes et al., 2011; Chen et al., 2014).

Despite the difference in the number of reports available for *C. neoformans* and *C. gattii* in the Fungal MLST Database (*n* = 487 for *C. neoformans* and *n* = 344 for *C. gattii*), preliminary analysis of the molecular targets available in the MLST scheme as an indicator of selection pressure (Cooper and Feil, 2004) revealed similar results for the two species. For example, the number of polymorphic sites, haplotype, and nucleotide diversity were similar for both individual genes and sequentially evaluated genes using concatenated sequences, with the exception of the number of haplotypes where the results were higher for *C. gattii* (despite a lower number of sequences). Two specific findings were of note: in the case of *C. neoformans*, the *IGS1* gene possessed the highest number of alleles (**Figure 3A**), a low percentage of sequence identity, high nucleotide diversity, and a high number of mutations compared with other genes (**Table 1**), which may be related to the function of this gene (i.e., a ribosomal RNA intergenic spacer) (Meyer et al., 2009). Previously, it has been shown that non-housekeeping genes could be subjected to strong diversifying or directional selection (Cooper and Feil, 2004). In the case of *C. gattii*, the *IGS1* gene shared similar characteristics with the equivalent gene in *C. neoformans*. The *SOD1* gene that presented the highest number of alleles (**Figure 3A**) and mutations (**Table 1**), which were unexpected characteristics for a housekeeping gene, thereby indicating that this gene may be subject to selection pressures that differ from those of other housekeeping genes (Cooper and Feil, 2004).

The evaluation of the utility of the MLST scheme for the two species showed that in general the genes present a high DP (>0.8 in all cases). However, in the case of TE, the results were heterogeneous among the genes (**Table 2**), with *IGS1* having the lowest value among the two species (0.173 and 0.253 for *C. neoformans* and *C. gattii*, respectively), which reflects the hypervariable nature of this marker. Hypervariability may lead to overestimation of the number of genotypes and thus reduce the possibility of the generation of typing units. Additionally, the heterozygosity, and the sequencing of independent loci, favors the over estimation of STs and therefore limits the allocation of isolates to a given genotype; this has been reported for other diploid pathogens such as *Candida* (Alanio et al., 2017). Therefore, the variability of these two genes (*IGS1* and *SOD1*) may contribute to overestimation of the diversity of these species. It has also been shown that the inclusion of genes with such hypervariable characteristics may help to discriminate closely related clones, potentially providing insight into recent evolutionary events (Feil and Spratt, 2001).

**FIGURE 8:**
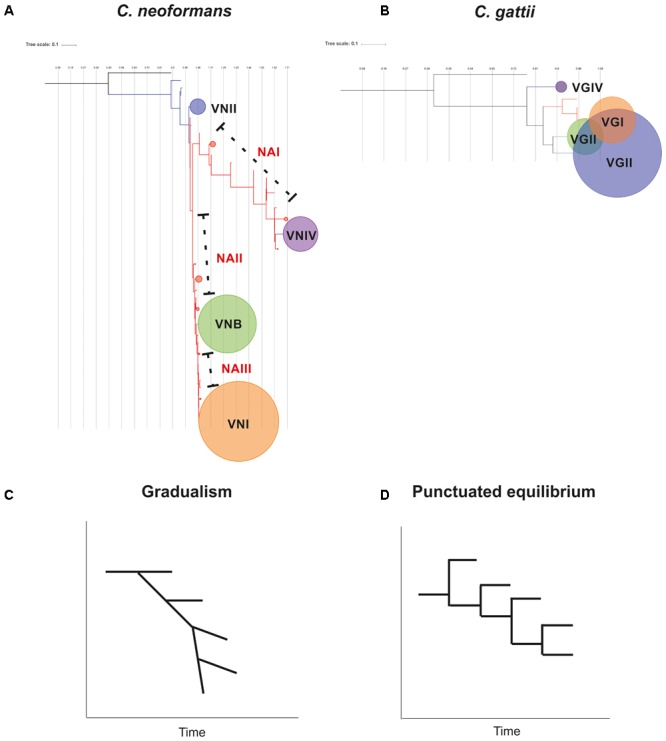
Hypothesis of the evolution of *Cryptococcus* complex members, based on phylogenetic relationships revealed by sequence analysis of the MLST scheme. The trees show the topology for *C. neoformans* **(A)** and *C. gattii* **(B)**. The circles represent the nodes where the molecular types of each species are grouped. The size of the circles is an indicator of the number of STs included in each cluster. A graphical representation of the structure of the phylogenetic tree is shown for *C. neoformans* **(C)** and *C. gattii* **(D)**, where the hypothesis of characteristic evolutionary models of gradualism and punctuated equilibrium are supported.

Phylogenetic analyzes of the concatenated sequences of the seven housekeeping genes in each species allowed for the elucidation of clustering patterns according to the four molecular types in each case (**Figure 4**). However, in the case of *C. neoformans*, some STs could not be assigned to any of the main clusters. These clusters were designated as NA I, II, and III according to their differential location within the phylogenetic tree, between each of the clusters. The topology identified for *C. neoformans* allowed us to propose the hypothesis that this species presents signatures of evolution by gradualism (as a continuous process in which big changes result from many cumulative small changes) (Singh, 2014). In this model, members of the VNII molecular type may represent the ancestor that underwent gradual evolutionary events to generate the NA groups and other molecular types (first VNIV, then VNB, and last VNI). **Figure 8A** shows this evolutionary profile, in which in the initial phylogeny the nodes that support the molecular types collapsed. The hypothesis of evolution by gradualism as applied to the topology of the phylogenetic tree is represented graphically in **Figure 8C**. In the case of *C. gattii*, phylogenetic reconstruction showed that molecular type 4 may have arisen as a result of divergence events that gave rise to well-established clusters (**Figure 8B**), coinciding with the principle of punctuated equilibrium (abrupt and rapid changes that give rise to well-differentiated clusters) (Singh, 2014). The application of this hypothesis on the data is plotted in **Figure 8D**. These differences in evolutionary patterns could be a further indicator of complex evolutionary histories resulting from the processes of adaptation to both natural environments and the colonization of hosts in the form of opportunistic infections, as previously reported (Desjardins et al., 2017).

Analysis of the population structure based on the algorithm eBURST revealed that in the case of *C. neoformans*, two of the CCs included the majority of STs (**Figure 5A** and Supplementary Table S2), whereas in the case of *C. gattii*, there was a greater number of CCs with a more homogeneous distribution (**Figure 5B** and Supplementary Table S3). These results agreed with the results of the phylogenetic reconstructions, confirming the close relationship between members of *C. neoformans* and a more defined population structure for *C. gattii*. Both the construction of phylogenetic networks (**Figure 6**), the estimated logarithm of probability using STRUCTURE (**Figure 7**), and the detection and characterization of recombination events and signals attributable to processes other than recombination (by RDP) supported the existence of genetic exchange events. Genetic exchange can occur between: (i) genotypes of the same lineage and can lead to mitochondrial hybrids, such as the hybrid between VGII- and VGIII-generated *in vitro* (Voelz et al., 2013) or (ii) between genotypes belonging to different species (*C. neoformans* and *C. gattii*), which can result in natural hybrids, which have been characterized through molecular analysis (Bovers et al., 2006, 2008b; Aminnejad et al., 2012; Farrer et al., 2015). The importance of such events has been documented by the adaptive evolution of fungal pathogens (Moran et al., 2011). Genetic exchange occurs widely in nature, even between prokaryotes and eukaryotes (Hirt et al., 2015; Sieber et al., 2017). Fungi are widely distributed in nature and there is therefore great opportunity for gene transfer to occur. Genetic exchange mechanisms include horizontal transfer, supernumerary chromosomes, and polyploidy, with some of these mechanisms being associated with virulence in fungi (Ma et al., 2010; Richards and Archibald, 2011; Richards et al., 2011). The abundant genetic diversity exhibited by *C. gattii* may be associated with the high genomic diversity that has been reported for its lineages, as well as the presence of both clonal populations and populations resulting from sexual recombination. All of this diversity could contribute to the generation of fitness advantages, the colonization of different types of populations (immunocompetent and immunosuppressed), and genomic rearrangements that ultimately benefit the adaptive capacity of the pathogen to new environments (D’Souza et al., 2011; Farrer et al., 2015, 2016; Feretzaki et al., 2016).

The association between MLST data and the virulence of pathogens has been demonstrated for *C. neoformans var. grubii* (Beale et al., 2015) and others prokaryotic microorganisms such as *Klebsiella pneumoniae* (Lin et al., 2016), *Streptococcus* (Chu et al., 2016; Emaneini et al., 2016), and *Staphylococcus* (Bouchami et al., 2016; Heijden et al., 2016), where the diversity of the population structure and evidence of genetic exchange(either as a result of point mutations or mobilization by horizontal gene transfer) are indicators of the success of these microorganisms as opportunistic pathogens (Smith et al., 2000). Similarly, in fungi, genetic level differences and expansion or contraction events in the genome relate to the survival of organisms in their niche environment and their pathogenic potential (Farrer et al., 2015). It is crucial to study diverse populations at the genomic level to reveal the existence of genetic alterations or rearrangements (such as point mutations and the presence/absence of gene duplication events) related to the mechanisms of pathogenesis that may indicate the likelihood of success of a particular genotype infection (Moran et al., 2011). This intra-taxa diversity together with the existence of intra- and inter-species hybrids can be considered an indirect indicator of genetic diversity. All of these processes may be the result of sexual reproduction events and recombination events resulting from the interaction between species or lineages that favor genetic exchange as a strategy to proliferate even under changing conditions such as those represented by human and animal hosts.

The evolutionary insights identified in this study could be the evidence of the effect of ecology on the species of *Cryptococcus* complex. The obtained phylogenetic trees, and their corresponding representation in the proposed evolutionary models (both gradualism and punctual equilibrium), show time-scale changes in the grouping, as a result of the accumulation of changes in the sequences of the constitutive genes analyzed (**Figure 8**). This phenomenon could occur in response to the trophic dynamics to which these pathogenic species have been exposed due to the variations in the ecological niches (either environmental or host), which could shape the pathogen evolution and described as a common event in different pathogens (Jackson, 2015). This pattern was recently reported for *Cryptococcus* complex (Vanhove et al., 2017). During the study of host–pathogen interactions, one of the most studied ecology driven processes is the effect of host immunity on genetic diversity in pathogens, in response to the simple process of interaction between species, in which the pathogen seeks to adapt to the new and adverse conditions that the host represents (Cobey, 2014). This series of evolutionary processes could be the basis of trade-off hypothesis of virulence, where the need to have an adequate environment for its survival and proliferation could lead to an adaptation to host conditions favoring host resistance or tolerance, which has been described for bacteria (Reding-Roman et al., 2017) and other species (Myers and Cory, 2016). The advent of genomic data will help to truly rule out the evolutionary pathways of *Cryptococcus* complex and how the ecology shapes their virulence and other biological traits.

## Conclusion

This work represents basic knowledge about the genetic diversity of species of the *Cryptococcus* complex, condensing for the first time analysis of MLST data (a set of data widely used and constantly enriched by the scientific community around the world). These efforts helped elucidating the evolutionary insights as the basis to understanding the differential impact of molecular types on the pathogenic characteristics of these fungi. Additionally, considering the already published population genomics studies (to elucidating the virulence factors, the inter- and intra-taxa diversity, and the evolutionary pathways from a more holistic way) and transcriptomics (to evaluate gene activation pathways during interaction with the host) will also be pivotal to identify the factors involved in the genesis of different clinical manifestations. An improved understanding of these fungal pathogens may lead to the identification of molecular targets for the development of tools for early diagnosis, the monitoring of infections, as well as the design of much-needed novel therapeutic strategies. These approaches may in the future contribute toward reducing the burden of morbidity and mortality associated with *Cryptococcus* fungal infection.

## Author Contributions

MM and MC conceived and designed the study, analyzed and interpreted the data, and wrote the paper. JR conceived and designed the study and revised the manuscript. All authors have reviewed and approved the manuscript.

## Conflict of Interest Statement

The authors declare that the research was conducted in the absence of any commercial or financial relationships that could be construed as a potential conflict of interest.

## Footnotes

1 http://mlst.mycologylab.org/

2 http://itol.embl.de

3 http://eburst.mlst.net/
