# Supplementary material for: Estimating the Intra-taxa Diversity, Population Genetic Structure, and Evolutionary Pathways of Cryptococcus neoformans and Cryptococcus gattii
Source: Front Genet. 2018 Apr 24;9:148. doi: 10.3389/fgene.2018.00148 (PMC5928140; doi:10.3389/fgene.2018.00148)
Supplement: Supplementary file 1 [file Presentation_1.PDF]

Supplementary Material

Estimating the intra-taxa diversity, population genetic structure and evolutionary pathways of *Cryptococcus neoformans* and *Cryptococcus gattii*

Marina Muñoz [1,2,3¶], Milena Camargo [2,4,5¶], Juan David Ramírez[1]*

[1]Universidad del Rosario, Facultad de Ciencias Naturales y Matemáticas, Programa de Biología, Grupo de Investigaciones Microbiológicas UR (GIMUR), Bogotá, Colombia

[2] Centro de Tecnología en Salud (CETESA), Upqua SAS, Bogotá, Colombia

[3] Posgrado Interfacultades Doctorado en Biotecnología, Facultad de Ciencias, Universidad Nacional de Colombia, Bogotá, Colombia

[4] Departamento de Biología Molecular e Inmunología, Fundación Instituto de Inmunología de Colombia (FIDIC), Bogotá, Colombia

[5] Doctorado en Ciencias Biomédicas, Universidad del Rosario, Bogotá, Colombia

[¶] These authors contributed equally to this work.

* **Correspondence:**

Corresponding author:

E-mail: juand.ramirez@urosario.edu.co (JDR)

Supplementary Material

Summary

The supplementary material includes 2 figures and 5 tables.

Supplementary information legends

Supplementary Figure S1. Phylogenetic networks for each marker in the MLST scheme (based on the neighbor–net algorithm) according to species. **A.** *Cryptococcus neoformans*. **B.** *Cryptococcus gattii*.

Supplementary Table S1. Diversity indexes related to the molecular types of *Cryptococcus* complex species.

Supplementary Table S2. Description of the STs and clonal complexes (CCs) for the structures observed in the eBURST diagram of *Cryptococcus neoformans.*

Supplementary Table S3. Description of the STs and clonal complexes (CCs) for the structures observed in the eBURST diagram of *Cryptococcus gattii*

Supplementary Figure S2. Recombination breakpoints matrix of *Cryptococcus* complex species. The recombination breakpoints are predicted via Recombination Detection Program (RDP). **A.** *Cryptococcus neoformans.* **B.** *Cryptococcus gattii.*

Supplementary Table S4. Characterization of recombination events for *Cryptococcus neoformans.*

Supplementary Table S5. Characterization of recombination events for *Cryptococcus gattii.*

Figure S1.

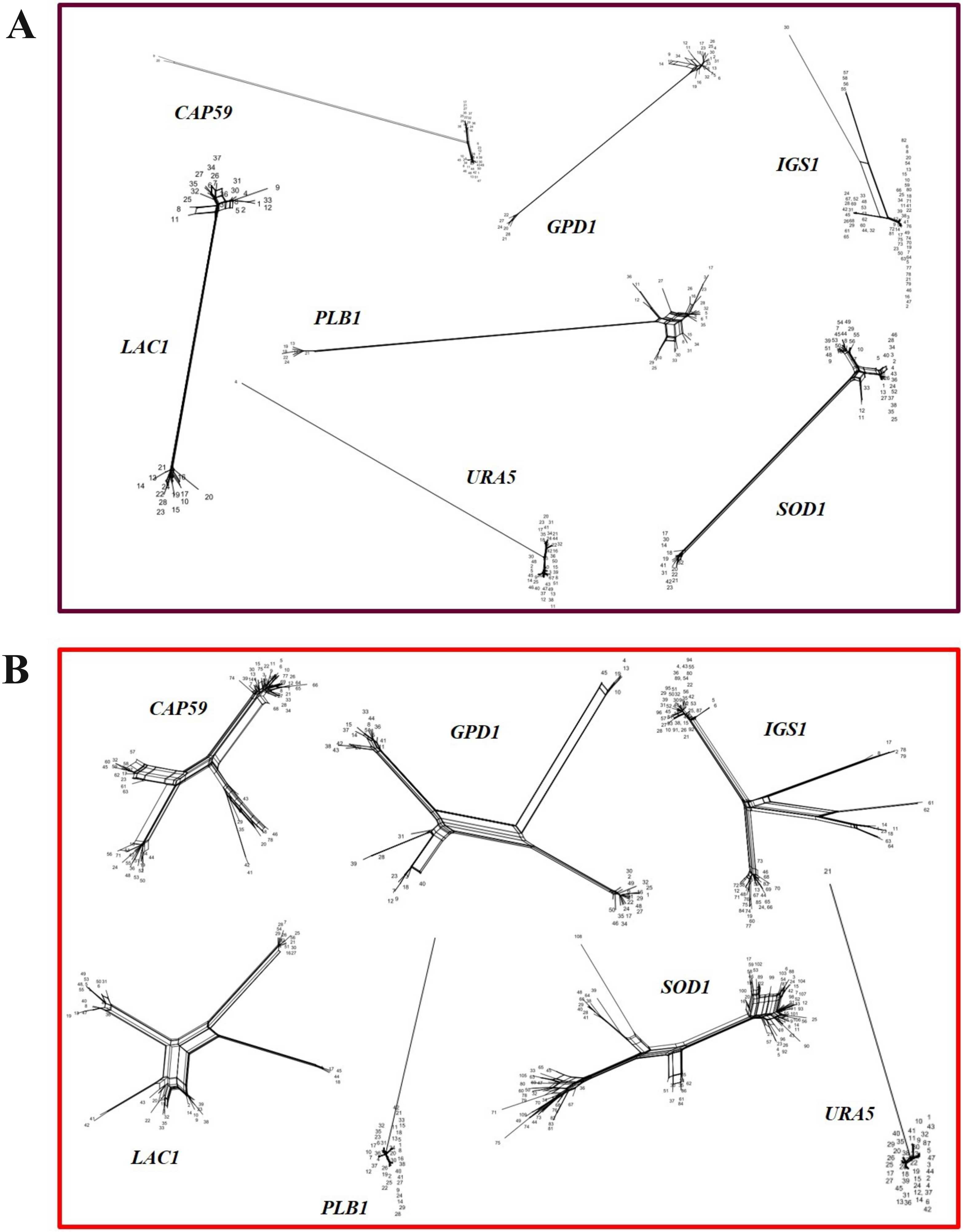
A
CAP59
GPD1
IGS1
LAC1
PLB1
URA5
SOD1
B
CAP59
GPD1
IGS1
LAC1
PLB1
SOD1
URA5

Table S1.

	Cryptococcus neoformans							*Cryptococcus gattii*			
	**VNI**	**VNII**	**VNIV**	**VNB**	**NAI**	**NAII**	**NAIII**	**VGI**	**VGII**	**VGIII**	**VGIV**
**Sequence length**	4.230	4.230	4.230	4.230	4.230	4.230	4.230	4.254	4.254	4.254	4.254
**Number of sequences used**	175	37	76	127	33	25	14	88	167	68	21
**Total number of sites (excluding sites with gaps / missing data)**	3.980	3.890	3.929	3.972	3.894	3.974	3.997	4.040	4.033	4.123	4.157
**Number of polymorphic (segregating) sites (S)**	112	341	95	120	370	93	32	455	207	341	41
**Haplotypes**	137	36	76	112	32	24	13	85	147	66	19
**Haplotype diversity (Hd)**	0.996	0.998	1.000	0.998	0.998	0.997	0.989	0.997	0.998	0.999	0.995
**Nucleotide diversity (Pi)**	0.002	0.023	0.003	0.003	0.037	0.004	0.002	0.011	0.004	0.007	0.002
**Number of mutations (Eta)**	113	349	97	123	372	93	32	472	213	354	41
**Theta (per site) from Eta**	0.004	0.021	0.005	0.005	0.023	0.006	0.002	0.022	0.009	0.017	0.002
**Average number of nucleotide differences (k)**	11.187	90.335	14.798	13.470	145.525	16.537	8.923	44.957	16.874	31.484	11.674
**Index of Association (IA)**	0.0267	0.2864	0.0299	0.1012	0.1538	0.1594	0.0991	0.2804	0.0441	0.1853	0.0025

Table S2.

C. neoformans

No. isolates = 487 | No. STs = 487 | No. re-samplings for bootstrapping = 1000
No. loci per isolate = 7 | No. identical loci for group def = 6 | No. groups = 37

Group 1: No. Isolates = 138 | No. STs = 138 | Predicted Founder = 5								
						Average	ST Bootstrap	
ST	FREQ	SLV	DLV	TLV	SAT	Distance	Group	Subgrp
5-I	1	20	9	37	71	3.41	74%	100%
31-I	1	15	22	33	67	3.37	22%	99%
23-I	1	15	18	14	90	4.02	18%	97%
2-I	1	14	21	13	89	3.97	14%	96%
93-I	1	14	10	31	82	3.95	11%	98%
174-I	1	13	35	47	42	2.91	9%	91%
32-I	1	12	27	28	70	3.48	3%	92%
67-I	1	11	27	10	89	3.94	0%	54%
63-I	1	11	19	29	78	3.54	2%	91%
311-ND	1	10	22	23	82	3.79	0%	18%
214-I	1	10	22	15	90	4.06	0%	23%
213-I	1	10	21	19	87	3.90	0%	17%
3-I	1	9	34	48	46	3.02	0%	48%
1-I	1	9	22	28	78	3.57	0%	53%
6-I	1	9	17	39	72	3.46	0%	81%
4-I	1	9	7	33	88	3.72	0%	77%
57-I	1	8	32	57	40	2.94	0%	25%
61-I	1	8	25	21	83	3.62	0%	27%
65-I	1	8	23	16	90	4.08	0%	0%
229-I	1	8	23	15	91	4.08	0%	0%
66-I	1	8	23	15	91	4.09	0%	0%
37-I	1	8	19	37	73	3.53	0%	38%
81-I	1	7	43	36	51	3.02	0%	17%
289-I	1	7	25	56	49	3.09	0%	33%
212-I	1	7	24	40	66	3.39	0%	4%
193-I	1	7	21	13	96	3.96	0%	35%
58-I	1	7	19	40	71	3.49	0%	31%
188-I	1	7	17	38	75	3.57	0%	19%
230-I	1	6	23	34	74	3.50	0%	0%
77-I	1	6	22	37	72	3.54	0%	6%
191-I	1	6	21	47	63	3.48	0%	16%
338-I	1	6	20	33	78	3.86	0%	6%
87-I	1	6	11	34	86	3.68	0%	71%
175-I	1	5	29	50	53	3.17	0%	3%
234-ND	1	5	27	53	52	3.13	0%	1%
364-ND	1	5	26	55	51	3.16	0%	12%
194-I	1	5	24	24	84	3.87	0%	11%
312-I	1	5	22	37	73	3.56	0%	2%
192-I	1	5	22	37	73	3.56	0%	2%
76-I	1	5	22	37	73	3.56	0%	2%
324-ND	1	5	21	29	82	3.68	0%	0%
202-I	1	5	19	23	90	3.81	0%	4%
92-I	1	5	18	31	83	4.05	0%	1%
91-I	1	5	18	31	83	4.05	0%	1%
292-ND	1	5	18	31	83	4.05	0%	1%

Table S3.

C. gattii

No. isolates = 344 | No. STs = 344 | No. re-samplings for bootstrapping = 1000
No. loci per isolate = 7 | No. identical loci for group def = 6 | No. groups = 50

Group 1: No. Isolates = 22 | No. STs = 22 | Predicted Founder = 39								
						Average	ST Bootstrap	
ST	FREQ	SLV	DLV	TLV	SAT	Distance	Group	Subgrp
51-I	1	10	10	1	0	1.57	76%	100%
154-I	1	9	11	1	0	1.61	61%	98%
57-I	1	4	15	2	0	1.90	2%	18%
159-I	1	4	9	7	1	2.23	3%	5%
102-I	1	4	9	7	1	2.23	4%	12%
188-I	1	4	8	8	1	2.28	5%	8%
149-I	1	3	11	7	0	2.19	1%	0%
63-I	1	3	11	7	0	2.19	0%	0%
356-ND	1	3	10	7	1	2.28	0%	0%
190-I	1	3	9	8	1	2.33	0%	0%
52-I	1	3	9	8	1	2.33	0%	0%
334-I	1	2	7	11	1	2.52	0%	0%
327-I	1	1	12	8	0	2.33	0%	0%
191-I	1	1	11	9	0	2.38	0%	0%
108-I	1	1	11	9	0	2.38	0%	0%
186-I	1	1	10	9	1	2.47	0%	0%
257-I	1	1	9	10	1	2.52	0%	0%
329-I	1	1	9	10	1	2.52	0%	0%
262-I	1	1	8	11	1	2.57	0%	0%
187-I	1	1	6	14	0	2.61	0%	0%
202-I	1	1	5	9	6	2.95	0%	0%
189-I	1	1	4	9	7	3.04	0%	0%

Group 2: No. Isolates = 13 | No. STs = 13 | Predicted Founder = 144								
						Average	ST Bootstrap	
ST	FREQ	SLV	DLV	TLV	SAT	Distance	Group	Subgrp
156-I	1	8	3	1	0	1.41	98%	99%
197-I	1	4	7	1	0	1.75	25%	11%
200-I	1	3	7	2	0	1.91	0%	0%
199-I	1	3	7	2	0	1.91	0%	0%
103-I	1	3	6	2	1	2.08	9%	0%
194-I	1	2	7	2	1	2.16	0%	0%
196-I	1	2	7	2	1	2.16	0%	0%
56-I	1	2	4	6	0	2.33	2%	0%
229-I	1	1	9	2	0	2.08	0%	0%
147-I	1	1	7	3	1	2.33	0%	0%
198-I	1	1	5	4	2	2.58	0%	0%
234-I	1	1	2	7	2	2.83	0%	0%
205-I	1	1	1	4	6	3.25	0%	0%

Group 3: No. Isolates = 12 | No. STs = 12 | Predicted Founder = 13

ST	FREQ	SLV	DLV	TLV	SAT	Average Distance	ST Bootstrap Group	Subgrp
20-II	1	11	0	0	0	1.0	100%	100%
267-I	1	4	7	0	0	1.63	5%	11%
246-II	1	4	7	0	0	1.63	5%	11%
245-II	1	4	7	0	0	1.63	9%	11%
244-II	1	4	7	0	0	1.63	14%	11%
252-II	1	2	9	0	0	1.81	0%	0%
251-II	1	2	9	0	0	1.81	0%	0%
343-II	1	1	10	0	0	1.90	0%	0%
266-I	1	1	10	0	0	1.90	0%	0%
253-II	1	1	10	0	0	1.90	0%	0%
250-II	1	1	10	0	0	1.90	0%	0%
122-II	1	1	10	0	0	1.90	0%	0%

Group 4: No. Isolates = 9 | No. STs = 9 | Predicted Founder = 47

ST	FREQ	SLV	DLV	TLV	SAT	Average Distance	ST Bootstrap Group	Subgrp
59-III	1	5	3	0	0	1.37	82%	77%
138-III	1	3	4	1	0	1.75	17%	10%
145-III	1	3	4	1	0	1.75	18%	23%
78-III	1	3	4	1	0	1.75	16%	10%
146-III	1	2	5	1	0	1.87	0%	0%
116-III	1	2	5	1	0	1.87	0%	0%
144-III	1	2	3	3	0	2.12	1%	0%
115-III	1	1	4	3	0	2.25	0%	0%
73-III	1	1	4	3	0	2.25	0%	0%

Group 5: No. Isolates = 7 | No. STs = 7 | Predicted Founder = 152

ST	FREQ	SLV	DLV	TLV	SAT	Average Distance	ST Bootstrap Group	Subgrp
164-III	1	6	0	0	0	1.0	97%	96%
77-III	1	3	3	0	0	1.5	3%	1%
68-III	1	3	3	0	0	1.5	2%	1%
62-III	1	3	3	0	0	1.5	11%	1%
335-III	1	2	4	0	0	1.66	0%	0%
55-III	1	2	4	0	0	1.66	0%	0%
210-III	1	1	5	0	0	1.83	0%	0%

Group 6: No. Isolates = 6 | No. STs = 6 | Predicted Founder = 6

ST	FREQ	SLV	DLV	TLV	SAT	Average Distance	ST Bootstrap Group	Subgrp
7-II	1	3	2	0	0	1.4	50%	20%
278-II	1	3	1	1	0	1.6	40%	17%
30-II	1	2	2	1	0	1.8	1%	0%
168-II	1	2	2	1	0	1.8	10%	0%
124-II	1	1	2	1	1	2.4	0%	0%
17-II	1	1	1	2	1	2.6	0%	0%

Group 7: No. Isolates = 6 | No. STs = 6 | Predicted Founder = Multiple Candidates								
						Average	ST Bootstrap	
ST	FREQ	SLV	DLV	TLV	SAT	Distance	Group	Subgrp
80-III	1	3	2	0	0	1.4	42%	17%
79-III	1	3	2	0	0	1.4	47%	20%
82-III	1	2	3	0	0	1.6	2%	0%
81-III	1	2	2	1	0	1.8	12%	0%
209-III	1	1	3	1	0	2.0	0%	0%
65-III	1	1	2	2	0	2.2	0%	0%

Group 8: No. Isolates = 5 | No. STs = 5 | Predicted Founder = 224								
						Average	ST Bootstrap	
ST	FREQ	SLV	DLV	TLV	SAT	Distance	Group	Subgrp
236-I	1	4	0	0	0	1.0	89%	52%
330-I	1	2	2	0	0	1.5	1%	0%
270-I	1	2	2	0	0	1.5	2%	0%
261-I	1	1	3	0	0	1.75	0%	0%
249-I	1	1	3	0	0	1.75	0%	0%

Group 9: No. Isolates = 5 | No. STs = 5 | Predicted Founder = 15								
						Average	ST Bootstrap	
ST	FREQ	SLV	DLV	TLV	SAT	Distance	Group	Subgrp
25-II	1	4	0	0	0	1.0	66%	51%
323-II	1	3	1	0	0	1.25	10%	12%
321-II	1	3	1	0	0	1.25	13%	11%
37-II	1	3	1	0	0	1.25	23%	12%
43-II	1	1	3	0	0	1.75	0%	0%

Group 10: No. Isolates = 5 | No. STs = 5 | Predicted Founder = Multiple Candidates								
						Average	ST Bootstrap	
ST	FREQ	SLV	DLV	TLV	SAT	Distance	Group	Subgrp
143-III	1	2	2	0	0	1.5	23%	0%
89-III	1	2	2	0	0	1.5	19%	0%
139-III	1	2	2	0	0	1.5	28%	0%
87-III	1	1	3	0	0	1.75	0%	0%
72-III	1	1	3	0	0	1.75	0%	0%

Group 11: No. Isolates = 4 | No. STs = 4 | Predicted Founder = Multiple Candidates								
						Average	ST Bootstrap	
ST	FREQ	SLV	DLV	TLV	SAT	Distance	Group	Subgrp
325-II	1	2	1	0	0	1.33	25%	0%
40-II	1	2	1	0	0	1.33	25%	0%
337-II	1	1	1	1	0	2.0	0%	0%
313-II	1	1	1	1	0	2.0	0%	0%

Group 12: No. Isolates = 4 | No. STs = 4 | Predicted Founder = 4

ST	FREQ	SLV	DLV	TLV	SAT	Average Distance	ST Bootstrap Group	Subgrp
5-II	1	3	0	0	0	1.0	58%	10%
288-II	1	2	1	0	0	1.33	4%	0%
265-I	1	2	1	0	0	1.33	9%	0%
296-II	1	1	2	0	0	1.66	0%	0%

Group 13: No. Isolates = 4 | No. STs = 4 | Predicted Founder = Multiple Candidates

ST	FREQ	SLV	DLV	TLV	SAT	Average Distance	ST Bootstrap Group	Subgrp
333-I	1	2	1	0	0	1.33	24%	0%
83-III	1	2	1	0	0	1.33	28%	0%
84-III	1	1	1	1	0	2.0	0%	0%
61-III	1	1	1	1	0	2.0	0%	0%

Group 14: No. Isolates = 4 | No. STs = 4 | Predicted Founder = 14

ST	FREQ	SLV	DLV	TLV	SAT	Average Distance	ST Bootstrap Group	Subgrp
21-III	1	3	0	0	0	1.0	57%	10%
255-II	1	2	1	0	0	1.33	5%	0%
254-II	1	2	1	0	0	1.33	11%	0%
260-II	1	1	2	0	0	1.66	0%	0%

Group 15: No. Isolates = 4 | No. STs = 4 | Predicted Founder = 94

ST	FREQ	SLV	DLV	TLV	SAT	Average Distance	ST Bootstrap Group	Subgrp
106-I	1	3	0	0	0	1.0	63%	12%
259-I	1	2	1	0	0	1.33	4%	0%
226-I	1	2	1	0	0	1.33	10%	0%
151-I	1	1	2	0	0	1.66	0%	0%

Group 16: No. Isolates = 4 | No. STs = 4 | Predicted Founder = 12

ST	FREQ	SLV	DLV	TLV	SAT	Average Distance	ST Bootstrap Group	Subgrp
19-II	1	3	0	0	0	1.0	59%	9%
131-II	1	2	1	0	0	1.33	4%	0%
18-II	1	2	1	0	0	1.33	9%	0%
167-II	1	1	2	0	0	1.66	0%	0%

Group 17: No. Isolates = 3 | No. STs = 3 | Predicted Founder = Multiple Candidates

ST	FREQ	SLV	DLV	TLV	SAT	Average Distance	ST Bootstrap Group	Subgrp
295-II	1	2	0	0	0	1.0	7%	0%
137-II	1	2	0	0	0	1.0	6%	0%
1-II	1	2	0	0	0	1.0	18%	0%

Group 18: No. Isolates = 3 | No. STs = 3 | Predicted Founder = Multiple Candidates

ST	FREQ	SLV	DLV	TLV	SAT	Average Distance	ST Bootstrap Group	Subgrp
277-II	1	2	0	0	0	1.0	8%	0%
132-II	1	2	0	0	0	1.0	7%	0%
26-II	1	2	0	0	0	1.0	13%	0%

Group 19: No. Isolates = 3 | No. STs = 3 | Predicted Founder = 218

ST	FREQ	SLV	DLV	TLV	SAT	Average Distance	ST Bootstrap Group	Subgrp
230-I	1	2	0	0	0	1.0	30%	0%
271-I	1	1	1	0	0	1.5	0%	0%
231-I	1	1	1	0	0	1.5	0%	0%

Group 20: No. Isolates = 3 | No. STs = 3 | Predicted Founder = Multiple Candidates

ST	FREQ	SLV	DLV	TLV	SAT	Average Distance	ST Bootstrap Group	Subgrp
269-I	1	2	0	0	0	1.0	7%	0%
162-I	1	2	0	0	0	1.0	6%	0%
111-I	1	2	0	0	0	1.0	19%	0%

Group 21: No. Isolates = 3 | No. STs = 3 | Predicted Founder = 88

ST	FREQ	SLV	DLV	TLV	SAT	Average Distance	ST Bootstrap Group	Subgrp
100-III	1	2	0	0	0	1.0	28%	0%
223-III	1	1	1	0	0	1.5	0%	0%
101-III	1	1	1	0	0	1.5	0%	0%

Group 22: No. Isolates = 3 | No. STs = 3 | Predicted Founder = Multiple Candidates

ST	FREQ	SLV	DLV	TLV	SAT	Average Distance	ST Bootstrap Group	Subgrp
171-II	1	2	0	0	0	1.0	6%	0%
33-II	1	2	0	0	0	1.0	10%	0%
32-II	1	2	0	0	0	1.0	14%	0%

Group 23: No. Isolates = 3 | No. STs = 3 | Predicted Founder = 157

ST	FREQ	SLV	DLV	TLV	SAT	Average Distance	ST Bootstrap Group	Subgrp
169-II	1	2	0	0	0	1.0	31%	0%
170-II	1	1	1	0	0	1.5	0%	0%
50-II	1	1	1	0	0	1.5	0%	0%

Group 24: No. Isolates = 3 | No. STs = 3 | Predicted Founder = Multiple Candidates

ST	FREQ	SLV	DLV	TLV	SAT	Average Distance	ST Bootstrap Group	Subgrp
97-III	1	2	0	0	0	1.0	6%	0%
96-III	1	2	0	0	0	1.0	6%	0%
90-III	1	2	0	0	0	1.0	16%	0%

Group 25: No. Isolates = 3 | No. STs = 3 | Predicted Founder = Multiple Candidates

ST	FREQ	SLV	DLV	TLV	SAT	Average Distance	ST Bootstrap Group	Subgrp
88-II	1	2	0	0	0	1.0	8%	0%
49-II	1	2	0	0	0	1.0	7%	0%
6-II	1	2	0	0	0	1.0	15%	0%

Group 26: No. Isolates = 2 | No. STs = 2 | Predicted Founder = None

ST	FREQ	SLV	DLV	TLV	SAT	Distance
346-II	1	1	0	0	0	1.0
46-II	1	1	0	0	0	1.0

Group 27: No. Isolates = 2 | No. STs = 2 | Predicted Founder = None

ST	FREQ	SLV	DLV	TLV	SAT	Distance
302-II	1	1	0	0	0	1.0
38-II	1	1	0	0	0	1.0

Group 28: No. Isolates = 2 | No. STs = 2 | Predicted Founder = None

ST	FREQ	SLV	DLV	TLV	SAT	Distance
286-II	1	1	0	0	0	1.0
357-II	1	1	0	0	0	1.0

Group 29: No. Isolates = 2 | No. STs = 2 | Predicted Founder = None

ST	FREQ	SLV	DLV	TLV	SAT	Distance
319-I	1	1	0	0	0	1.0
157-I	1	1	0	0	0	1.0

Group 30: No. Isolates = 2 | No. STs = 2 | Predicted Founder = None

ST	FREQ	SLV	DLV	TLV	SAT	Distance
281-II	1	1	0	0	0	1.0
280-II	1	1	0	0	0	1.0

Group 31: No. Isolates = 2 | No. STs = 2 | Predicted Founder = None

ST	FREQ	SLV	DLV	TLV	SAT	Distance
316-II	1	1	0	0	0	1.0
135-II	1	1	0	0	0	1.0

Group 32: No. Isolates = 2 | No. STs = 2 | Predicted Founder = None

ST	FREQ	SLV	DLV	TLV	SAT	Distance
243-IV	1	1	0	0	0	1.0
207-IV	1	1	0	0	0	1.0

Group 33: No. Isolates = 2 | No. STs = 2 | Predicted Founder = None

ST	FREQ	SLV	DLV	TLV	SAT	Distance
203-III	1	1	0	0	0	1.0
60-III	1	1	0	0	0	1.0

Group 34: No. Isolates = 2 | No. STs = 2 | Predicted Founder = None

ST	FREQ	SLV	DLV	TLV	SAT	Distance
233-I	1	1	0	0	0	1.0
232-I	1	1	0	0	0	1.0

Group 35: No. Isolates = 2 | No. STs = 2 | Predicted Founder = None

ST	FREQ	SLV	DLV	TLV	SAT	Distance
193-I	1	1	0	0	0	1.0
58-I	1	1	0	0	0	1.0

Group 36: No. Isolates = 2 | No. STs = 2 | Predicted Founder = None

ST	FREQ	SLV	DLV	TLV	SAT	Distance
224-IV	1	1	0	0	0	1.0
105-IV	1	1	0	0	0	1.0

Group 37: No. Isolates = 2 | No. STs = 2 | Predicted Founder = None

ST	FREQ	SLV	DLV	TLV	SAT	Distance
182-II	1	1	0	0	0	1.0
181-II	1	1	0	0	0	1.0

Group 38: No. Isolates = 2 | No. STs = 2 | Predicted Founder = None

ST	FREQ	SLV	DLV	TLV	SAT	Distance
218-I	1	1	0	0	0	1.0
212-I	1	1	0	0	0	1.0

Group 39: No. Isolates = 2 | No. STs = 2 | Predicted Founder = None

ST	FREQ	SLV	DLV	TLV	SAT	Distance
180-II	1	1	0	0	0	1.0
179-II	1	1	0	0	0	1.0

Group 40: No. Isolates = 2 | No. STs = 2 | Predicted Founder = None

ST	FREQ	SLV	DLV	TLV	SAT	Distance
178-II	1	1	0	0	0	1.0
177-II	1	1	0	0	0	1.0

Group 41: No. Isolates = 2 | No. STs = 2 | Predicted Founder = None

ST	FREQ	SLV	DLV	TLV	SAT	Distance
155-IV	1	1	0	0	0	1.0
150-IV	1	1	0	0	0	1.0

Group 42: No. Isolates = 2 | No. STs = 2 | Predicted Founder = None

ST	FREQ	SLV	DLV	TLV	SAT	Distance
148-III	1	1	0	0	0	1.0
93-III	1	1	0	0	0	1.0

Group 43: No. Isolates = 2 | No. STs = 2 | Predicted Founder = None

ST	FREQ	SLV	DLV	TLV	SAT	Distance
142-III	1	1	0	0	0	1.0
66-III	1	1	0	0	0	1.0

Group 44: No. Isolates = 2 | No. STs = 2 | Predicted Founder = None

ST	FREQ	SLV	DLV	TLV	SAT	Distance
136-II	1	1	0	0	0	1.0
308-II	1	1	0	0	0	1.0

Group 45: No. Isolates = 2 | No. STs = 2 | Predicted Founder = None

ST	FREQ	SLV	DLV	TLV	SAT	Distance
134-II	1	1	0	0	0	1.0
352-II	1	1	0	0	0	1.0

Group 46: No. Isolates = 2 | No. STs = 2 | Predicted Founder = None

ST	FREQ	SLV	DLV	TLV	SAT	Distance
130-II	1	1	0	0	0	1.0
35-II	1	1	0	0	0	1.0

Group 47: No. Isolates = 2 | No. STs = 2 | Predicted Founder = None

ST	FREQ	SLV	DLV	TLV	SAT	Distance
110-I	1	1	0	0	0	1.0
362-I	1	1	0	0	0	1.0

Group 48: No. Isolates = 2 | No. STs = 2 | Predicted Founder = None

ST	FREQ	SLV	DLV	TLV	SAT	Distance
95-III	1	1	0	0	0	1.0
94-III	1	1	0	0	0	1.0

Group 49: No. Isolates = 2 | No. STs = 2 | Predicted Founder = None

ST	FREQ	SLV	DLV	TLV	SAT	Distance
92-III	1	1	0	0	0	1.0
91-III	1	1	0	0	0	1.0

Group 50: No. Isolates = 2 | No. STs = 2 | Predicted Founder = None

ST	FREQ	SLV	DLV	TLV	SAT	Distance
354-II	1	1	0	0	0	1.0
312-II	1	1	0	0	0	1.0

Singletons: size 153

347-II
345-II
344-II
342-II
341-II
340-II
338-II
305-II
304-II
303-II
301-II
300-II
299-II
298-II
297-II
332-I
331-I
328-II
326-III
293-II
292-II
290-II
289-II
287-II
283-II
282-II
324-II
322-II
320-III
318-I
317-II
279-II
276-II
275-II
274-II
273-II
272-II
268-I
264-I
263-IV
258-II
256-I
248-II
247-II
211-I
242-IV
208-I
206-IV
204-I
241-IV
240-IV
239-IV
238-IV
237-IV
235-I
201-I
195-I
192-I
228-I
227-I
225-I
222-I
185-II
184-II
183-II
221-I
220-I
219-I
217-I
216-I
215-I
214-I
213-I
176-II
175-II
174-II
173-II

172-II
166-III
165-III
163-III
161-I
160-IV
158-IV
153-I
152-I
141-III
140-III
133-II
129-II
128-II
127-II
126-II
125-II
123-II
121-II
120-II
119-II
118-III
117-III
114-III
113-III
112-III
109-I
107-IV
104-IV
99-III
98-III
86-III
85-III
76-III
75-III
74-III
71-II
70-IV
69-IV
67-III
64-III
54-I
53-I
48-II
47-II
45-II
44-II
42-II
39-II
34-II
31-II

12-II
10-II
8-II
361-II
360-II
4-II
359-II
3-II
358-II
29-II
28-II
27-II
355-II
353-II
351-ND
350-II
349-II
315-II
348-II
314-II
311-II
310-II
309-II
307-II
306-II

85-I	1	5	17	16	99	4.05	0%	4%
374-I	1	5	17	16	99	4.05	0%	5%
360-I	1	5	17	16	99	4.05	0%	4%
341-I	1	5	14	38	80	3.64	0%	0%
189-I	1	5	11	35	86	3.73	0%	0%
82-I	1	5	10	35	87	3.75	0%	0%
80-I	1	5	10	35	87	3.71	0%	51%
53-I	1	4	36	42	55	3.30	0%	0%
38-I	1	4	31	33	69	3.40	0%	0%
187-I	1	4	29	20	84	3.85	0%	0%
299-I	1	4	28	32	73	3.49	0%	0%
484-I	1	4	26	26	81	3.70	0%	0%
56-I	1	4	25	32	76	3.57	0%	0%
337-I	1	4	23	38	72	3.56	0%	1%
39-I	1	4	23	35	75	3.66	0%	0%
75-I	1	4	23	35	75	3.66	0%	0%
291-ND	1	4	22	43	68	3.45	0%	0%
226-I	1	4	20	14	99	3.86	0%	16%
95-I	1	4	17	27	89	4.12	0%	3%
94-I	1	4	15	29	89	4.13	0%	0%
264-I	1	4	14	55	64	3.37	0%	3%
68-I	1	4	12	22	99	4.40	0%	5%
483-I	1	3	26	49	59	3.26	0%	0%
186-I	1	3	23	36	75	3.59	0%	0%
359-I	1	3	23	36	75	3.59	0%	0%
237-ND	1	3	22	45	67	3.52	0%	0%
197-I	1	3	21	59	54	3.21	0%	0%
198-I	1	3	20	43	71	3.56	0%	0%
79-I	1	3	19	19	96	4.06	0%	0%
298-I	1	3	19	19	96	4.06	0%	0%
204-I	1	3	18	8	108	4.28	0%	0%
203-I	1	3	18	8	108	4.29	0%	0%
296-II	1	3	18	8	108	4.29	0%	0%
314-ND	1	3	17	22	95	4.22	0%	0%
376-ND	1	3	17	22	95	4.23	0%	0%
137-I	1	3	15	19	100	4.10	0%	1%
307-ND	1	3	14	17	103	4.49	0%	0%
47-I	1	3	13	18	103	4.59	0%	0%
54-I	1	3	11	35	88	3.81	0%	1%
248-ND	1	3	11	24	99	4.0	0%	0%
13-I	1	3	10	40	84	3.72	0%	13%
370-ND	1	3	9	37	88	3.78	0%	0%
89-I	1	3	7	22	105	4.32	0%	2%
25-B	1	2	28	38	69	3.51	0%	0%
297-I	1	2	23	27	85	3.94	0%	0%
331-ND	1	2	22	59	54	3.43	0%	0%
319-ND	1	2	21	22	92	4.10	0%	0%
310-ND	1	2	20	9	106	4.10	0%	0%
265-ND	1	2	18	30	87	3.75	0%	0%
83-I	1	2	17	47	71	3.56	0%	0%
195-I	1	2	17	28	90	4.06	0%	0%
246-ND	1	2	17	19	99	4.85	0%	0%
250-I	1	2	17	17	101	4.90	0%	0%
103-I	1	2	17	17	101	4.90	0%	0%
236-ND	1	2	16	38	81	3.78	0%	0%

340-I	1	2	16	25	94	4.19	0%	0%
290-I	1	2	15	23	97	4.81	0%	0%
238-ND	1	2	14	31	90	3.89	0%	0%
247-ND	1	2	14	28	93	4.05	0%	0%
339-I	1	2	13	39	83	4.00	0%	0%
199-I	1	2	13	36	86	3.88	0%	0%
59-I	1	2	12	39	84	4.05	0%	0%
268-I	1	2	12	20	103	4.42	0%	0%
64-I	1	2	12	20	103	4.42	0%	0%
138-I	1	2	11	29	95	4.00	0%	0%
217-I	1	2	11	25	99	4.03	0%	0%
141-I	1	2	10	37	88	3.81	0%	0%
104-I	1	2	10	20	105	4.18	0%	0%
22-I	1	2	9	41	85	3.75	0%	0%
216-I	1	2	9	28	98	4.13	0%	0%
377-I	1	2	8	22	105	4.33	0%	0%
139-I	1	2	6	22	107	4.36	0%	0%
378-I	1	2	6	22	107	4.36	0%	0%
34-I	1	2	3	26	106	4.36	0%	0%
323-I	1	1	16	30	90	4.05	0%	0%
177-I	1	1	16	12	108	4.50	0%	0%
78-I	1	1	15	32	89	3.89	0%	0%
176-I	1	1	15	27	94	3.94	0%	0%
55-I	1	1	14	49	73	3.45	0%	0%
185-I	1	1	14	23	99	4.26	0%	0%
215-I	1	1	14	21	101	4.93	0%	0%
190-I	1	1	13	21	102	4.18	0%	0%
211-I	1	1	13	21	102	4.44	0%	0%
301-I	1	1	13	11	112	4.83	0%	0%
62-I	1	1	12	23	101	4.50	0%	0%
306-ND	1	1	8	20	108	4.16	0%	0%
300-I	1	1	7	28	101	3.97	0%	0%
333-ND	1	1	6	28	102	4.04	0%	0%
329-ND	1	1	6	17	113	4.74	0%	0%
257-ND	1	1	5	28	103	4.37	0%	0%
88-I	1	1	5	11	120	4.56	0%	0%
84-I	1	1	3	25	108	4.20	0%	0%
239-ND	1	1	1	25	110	4.18	0%	0%

Group 2: No. Isolates = 23 | No. STs = 23 | Predicted Founder = 150

ST	FREQ	SLV	DLV	TLV	SAT	Average Distance	ST Bootstrap Group	Subgrp
150-I	1	6	4	7	5	2.54	63%	85%
415-B	1	4	8	5	5	2.54	38%	51%
418-B	1	4	7	8	3	2.45	15%	21%
427-B	1	4	6	4	8	2.81	16%	32%
14-B	1	3	10	5	4	2.45	6%	15%
419-B	1	3	7	6	6	2.72	4%	11%
425-B	1	3	5	8	6	2.81	6%	10%
441-B	1	3	5	7	7	2.86	0%	1%
440-B	1	3	5	7	7	2.86	0%	1%
148-I	1	3	4	9	6	2.81	4%	22%
152-I	1	2	6	8	6	2.90	0%	0%
20-B	1	2	6	4	10	3.13	0%	0%
436-B	1	2	5	7	8	3.09	0%	0%
453-B	1	2	4	7	9	3.22	0%	0%
392-B	1	2	3	7	10	3.40	0%	0%
431-B	1	1	9	7	5	2.81	0%	0%
151-I	1	1	9	7	5	2.77	0%	0%
390-B	1	1	4	6	11	3.40	0%	0%
416-B	1	1	3	7	11	3.31	0%	0%
395-B	1	1	2	10	9	3.40	0%	0%
452-B	1	1	2	9	10	3.5	0%	0%
420-B	1	1	2	7	12	3.68	0%	0%
394-B	1	1	2	6	13	3.59	0%	0%

Group 3: No. Isolates = 10 | No. STs = 10 | Predicted Founder = 245

ST	FREQ	SLV	DLV	TLV	SAT	Average Distance	ST Bootstrap Group	Subgrp
245-ND	1	5	1	3	0	1.77	81%	79%
18-B	1	3	5	1	0	1.77	17%	3%
145-I	1	3	3	3	0	2.0	0%	3%
375-ND	1	3	3	3	0	2.0	1%	3%
227-IV	1	2	7	0	0	1.77	3%	0%
316-ND	1	2	6	1	0	1.88	17%	0%
16-B	1	2	3	3	1	2.33	12%	0%
228-IV	1	2	2	4	1	2.44	3%	0%
48-I	1	1	4	3	1	2.44	0%	0%
332-ND	1	1	4	1	3	2.66	0%	0%

Group 4: No. Isolates = 7 | No. STs = 7 | Predicted Founder = 69

ST	FREQ	SLV	DLV	TLV	SAT	Average Distance	ST Bootstrap Group	Subgrp
69-I	1	6	0	0	0	1.0	99%	94%
328-ND	1	2	4	0	0	1.66	0%	0%
105-I	1	2	4	0	0	1.66	0%	0%
72-I	1	2	4	0	0	1.66	0%	0%
71-I	1	2	4	0	0	1.66	0%	0%
218-I	1	1	5	0	0	1.83	0%	0%
369-ND	1	1	5	0	0	1.83	0%	0%

Group 5: No. Isolates = 7 | No. STs = 7 | Predicted Founder = 449

						Average	ST Bootstrap	
ST	FREQ	SLV	DLV	TLV	SAT	Distance	Group	Subgrp
449-I	1	4	1	1	0	1.5	67%	49%
330-ND	1	3	3	0	0	1.5	41%	14%
450-I	1	2	3	1	0	1.83	0%	0%
73-I	1	2	2	2	0	2.0	7%	0%
21-I	1	1	4	1	0	2.0	0%	0%
74-I	1	1	3	1	1	2.33	0%	0%
24-I	1	1	2	2	1	2.5	0%	0%

Group 6: No. Isolates = 6 | No. STs = 6 | Predicted Founder = 135

						Average	ST Bootstrap	
ST	FREQ	SLV	DLV	TLV	SAT	Distance	Group	Subgrp
135-IV	1	5	0	0	0	1.0	97%	81%
136-IV	1	2	3	0	0	1.6	0%	0%
349-ND	1	2	3	0	0	1.6	1%	0%
282-IV	1	1	4	0	0	1.8	0%	0%
251-IV	1	1	4	0	0	1.8	0%	0%
348-ND	1	1	4	0	0	1.8	0%	0%

Group 7: No. Isolates = 6 | No. STs = 6 | Predicted Founder = 304

						Average	ST Bootstrap	
ST	FREQ	SLV	DLV	TLV	SAT	Distance	Group	Subgrp
304-ND	1	4	1	0	0	1.2	73%	52%
9-B	1	3	2	0	0	1.4	37%	18%
303-ND	1	2	3	0	0	1.6	0%	0%
210-I	1	1	4	0	0	1.8	0%	0%
408-ND	1	1	3	1	0	2.0	0%	0%
410-ND	1	1	3	1	0	2.0	0%	0%

Group 8: No. Isolates = 6 | No. STs = 6 | Predicted Founder = 342

						Average	ST Bootstrap	
ST	FREQ	SLV	DLV	TLV	SAT	Distance	Group	Subgrp
342-ND	1	5	0	0	0	1.0	96%	80%
267-IV	1	2	3	0	0	1.6	0%	0%
343-ND	1	2	3	0	0	1.6	0%	0%
362-ND	1	2	3	0	0	1.6	0%	0%
344-ND	1	2	3	0	0	1.6	0%	0%
266-IV	1	1	4	0	0	1.8	0%	0%

Group 9: No. Isolates = 5 | No. STs = 5 | Predicted Founder = 121

						Average	ST Bootstrap	
ST	FREQ	SLV	DLV	TLV	SAT	Distance	Group	Subgrp
121-IV	1	3	1	0	0	1.25	61%	18%
162-IV	1	2	2	0	0	1.5	18%	0%
273-IV	1	1	2	1	0	2.0	0%	0%
122-IV	1	1	2	1	0	2.0	0%	0%
164-IV	1	1	1	2	0	2.25	0%	0%

Group 10: No. Isolates = 5 | No. STs = 5 | Predicted Founder = 40

ST	FREQ	SLV	DLV	TLV	SAT	Average Distance	ST Bootstrap Group	Subgrp
40-II	1	4	0	0	0	1.0	87%	51%
233-ND	1	2	2	0	0	1.5	1%	0%
100-II	1	2	2	0	0	1.5	4%	0%
209-II	1	1	3	0	0	1.75	0%	0%
207-II	1	1	3	0	0	1.75	0%	0%

Group 11: No. Isolates = 5 | No. STs = 5 | Predicted Founder = Multiple Candidates

ST	FREQ	SLV	DLV	TLV	SAT	Average Distance	ST Bootstrap Group	Subgrp
45-I	1	3	1	0	0	1.25	47%	17%
44-I	1	3	1	0	0	1.25	45%	14%
52-I	1	2	2	0	0	1.5	4%	0%
49-I	1	1	2	1	0	2.0	0%	0%
50-I	1	1	2	1	0	2.0	0%	0%

Group 12: No. Isolates = 4 | No. STs = 4 | Predicted Founder = Multiple Candidates

ST	FREQ	SLV	DLV	TLV	SAT	Average Distance	ST Bootstrap Group	Subgrp
334-II	1	2	1	0	0	1.33	27%	0%
172-II	1	2	1	0	0	1.33	24%	0%
243-ND	1	1	2	0	0	1.66	0%	0%
99-II	1	1	2	0	0	1.66	0%	0%

Group 13: No. Isolates = 4 | No. STs = 4 | Predicted Founder = 384

ST	FREQ	SLV	DLV	TLV	SAT	Average Distance	ST Bootstrap Group	Subgrp
384-ND	1	3	0	0	0	1.0	61%	13%
473-B	1	2	1	0	0	1.33	1%	0%
412-B	1	2	1	0	0	1.33	12%	0%
383-ND	1	1	2	0	0	1.66	0%	0%

Group 14: No. Isolates = 4 | No. STs = 4 | Predicted Founder = 90

ST	FREQ	SLV	DLV	TLV	SAT	Average Distance	ST Bootstrap Group	Subgrp
90-I	1	3	0	0	0	1.0	57%	13%
381-I	1	2	1	0	0	1.33	5%	0%
380-I	1	2	1	0	0	1.33	8%	0%
36-I	1	1	2	0	0	1.66	0%	0%

Group 15: No. Isolates = 3 | No. STs = 3 | Predicted Founder = 222

ST	FREQ	SLV	DLV	TLV	SAT	Average Distance	ST Bootstrap Group	Subgrp
222-II	1	2	0	0	0	1.0	26%	0%
221-II	1	1	1	0	0	1.5	0%	0%
42-II	1	1	1	0	0	1.5	0%	0%

Group 16: No. Isolates = 3 | No. STs = 3 | Predicted Founder = Multiple Candidates

ST	FREQ	SLV	DLV	TLV	SAT	Average Distance	ST Bootstrap Group	Subgrp
173-II	1	2	0	0	0	1.0	8%	0%
208-II	1	2	0	0	0	1.0	7%	0%
43-II	1	2	0	0	0	1.0	16%	0%

Group 17: No. Isolates = 3 | No. STs = 3 | Predicted Founder = 33

ST	FREQ	SLV	DLV	TLV	SAT	Average Distance	ST Bootstrap Group	Subgrp
33-B	1	2	0	0	0	1.0	31%	0%
144-I	1	1	1	0	0	1.5	0%	0%
398-B	1	1	1	0	0	1.5	0%	0%

Group 18: No. Isolates = 3 | No. STs = 3 | Predicted Founder = 481

ST	FREQ	SLV	DLV	TLV	SAT	Average Distance	ST Bootstrap Group	Subgrp
481-B	1	2	0	0	0	1.0	26%	0%
480-B	1	1	1	0	0	1.5	0%	0%
477-B	1	1	1	0	0	1.5	0%	0%

Group 19: No. Isolates = 3 | No. STs = 3 | Predicted Founder = Multiple Candidates

ST	FREQ	SLV	DLV	TLV	SAT	Average Distance	ST Bootstrap Group	Subgrp
457-B	1	2	0	0	0	1.0	7%	0%
456-B	1	2	0	0	0	1.0	8%	0%
455-B	1	2	0	0	0	1.0	16%	0%

Group 20: No. Isolates = 2 | No. STs = 2 | Predicted Founder = None

ST	FREQ	SLV	DLV	TLV	SAT	Distance
263-ND	1	1	0	0	0	1.0
224-I	1	1	0	0	0	1.0

Group 21: No. Isolates = 2 | No. STs = 2 | Predicted Founder = None

ST	FREQ	SLV	DLV	TLV	SAT	Distance
262-ND	1	1	0	0	0	1.0
223-I	1	1	0	0	0	1.0

Group 22: No. Isolates = 2 | No. STs = 2 | Predicted Founder = None

ST	FREQ	SLV	DLV	TLV	SAT	Distance
240-ND	1	1	0	0	0	1.0
205-I	1	1	0	0	0	1.0

Group 23: No. Isolates = 2 | No. STs = 2 | Predicted Founder = None

ST	FREQ	SLV	DLV	TLV	SAT	Distance
180-IV	1	1	0	0	0	1.0
128-IV	1	1	0	0	0	1.0

Group 24: No. Isolates = 2 | No. STs = 2 | Predicted Founder = None

ST	FREQ	SLV	DLV	TLV	SAT	Distance
160-IV	1	1	0	0	0	1.0
123-IV	1	1	0	0	0	1.0

Group 25: No. Isolates = 2 | No. STs = 2 | Predicted Founder = None

ST	FREQ	SLV	DLV	TLV	SAT	Distance
159-IV	1	1	0	0	0	1.0
293-ND	1	1	0	0	0	1.0

Group 26: No. Isolates = 2 | No. STs = 2 | Predicted Founder = None

ST	FREQ	SLV	DLV	TLV	SAT	Distance
156-IV	1	1	0	0	0	1.0
110-IV	1	1	0	0	0	1.0

Group 27: No. Isolates = 2 | No. STs = 2 | Predicted Founder = None

ST	FREQ	SLV	DLV	TLV	SAT	Distance
153-I	1	1	0	0	0	1.0
19-B	1	1	0	0	0	1.0

Group 28: No. Isolates = 2 | No. STs = 2 | Predicted Founder = None

ST	FREQ	SLV	DLV	TLV	SAT	Distance
146-I	1	1	0	0	0	1.0
12-B	1	1	0	0	0	1.0

Group 29: No. Isolates = 2 | No. STs = 2 | Predicted Founder = None

ST	FREQ	SLV	DLV	TLV	SAT	Distance
143-I	1	1	0	0	0	1.0
7-B	1	1	0	0	0	1.0

Group 30: No. Isolates = 2 | No. STs = 2 | Predicted Founder = None

ST	FREQ	SLV	DLV	TLV	SAT	Distance
124-IV	1	1	0	0	0	1.0
118-IV	1	1	0	0	0	1.0

Group 31: No. Isolates = 2 | No. STs = 2 | Predicted Founder = None

ST	FREQ	SLV	DLV	TLV	SAT	Distance
476-B	1	1	0	0	0	1.0
471-B	1	1	0	0	0	1.0

Group 32: No. Isolates = 2 | No. STs = 2 | Predicted Founder = None

ST	FREQ	SLV	DLV	TLV	SAT	Distance
443-B	1	1	0	0	0	1.0
423-B	1	1	0	0	0	1.0

Group 33: No. Isolates = 2 | No. STs = 2 | Predicted Founder = None

ST	FREQ	SLV	DLV	TLV	SAT	Distance
429-B	1	1	0	0	0	1.0
417-B	1	1	0	0	0	1.0

Group 34: No. Isolates = 2 | No. STs = 2 | Predicted Founder = None

ST	FREQ	SLV	DLV	TLV	SAT	Distance
51-I	1	1	0	0	0	1.0
46-I	1	1	0	0	0	1.0

Group 35: No. Isolates = 2 | No. STs = 2 | Predicted Founder = None

ST	FREQ	SLV	DLV	TLV	SAT	Distance
354-ND	1	1	0	0	0	1.0
353-ND	1	1	0	0	0	1.0

Group 36: No. Isolates = 2 | No. STs = 2 | Predicted Founder = None

ST	FREQ	SLV	DLV	TLV	SAT	Distance
352-ND	1	1	0	0	0	1.0
347-ND	1	1	0	0	0	1.0

Group 37: No. Isolates = 2 | No. STs = 2 | Predicted Founder = None

ST	FREQ	SLV	DLV	TLV	SAT	Distance
28-B	1	1	0	0	0	1.0
11-B	1	1	0	0	0	1.0

Singletons: size 206

327-ND
326-ND
325-ND
322-I
321-I
320-I
288-I
287-ND
286-IV
285-IV
284-IV
283-IV
281-IV
280-IV
318-I
317-ND
315-ND
313-II
279-IV
278-IV
277-IV
276-IV
275-IV

274-IV
272-IV
271-IV
270-IV
309-ND
308-ND
305-ND
302-ND
269-IV
261-ND
260-ND
259-ND
258-ND
256-ND
255-ND
254-ND
253-ND
252-IV
249-ND
244-ND
242-ND
241-ND
235-ND
232-ND

231-ND
196-I
225-I
220-II
184-I
183-I
182-I
181-IV
219-I
179-IV
178-IV
171-I
170-IV
206-I
169-IV
201-I
168-IV
200-I
167-IV
166-IV
165-IV
163-IV
161-IV
158-IV

157-IV
155-IV
154-IV
149-I
147-I
142-I
140-I
134-IV
133-IV
132-IV
131-IV
130-IV
129-IV
487-IV
486-IV
127-IV
485-II
126-IV
125-IV
482-B
120-IV
479-ND
478-B
119-IV
117-IV
475-B
116-IV
474-B
115-IV
114-IV
472-B
113-IV
112-IV
470-B
111-IV
469-ND
468-B
109-IV
467-II
108-IV
466-B
107-II
465-B
106-II
464-B
463-B
462-B
461-ND
102-B
460-B
101-II
98-II
97-II
96-II
459-B

458-B
454-B
451-B
86-I
448-ND
447-B
446-B
445-B
444-B
442-B
439-B
438-B
437-B
70-I
435-B
434-B
433-B
432-B
399-B
430-B
397-B
396-B
393-B
391-B
428-B
60-II
426-B
424-B
422-B
421-B
389-ND
388-ND
387-ND
386-ND
385-ND
382-ND
414-B
413-B
411-ND
379-I
373-ND
372-ND
371-ND
409-B
41-II
407-B
406-B
405-B
404-B
403-B
402-B
401-B
400-B
368-ND
367-ND

366-ND
365-ND
363-ND
361-ND
35-B
30-I
358-ND
357-ND
356-ND
355-ND
351-ND
350-ND
29-I
27-B
26-B
8-B
346-ND
345-ND
17-B
15-I
10-B
336-IV
335-IV
295-I
294-IV

Figure S2.

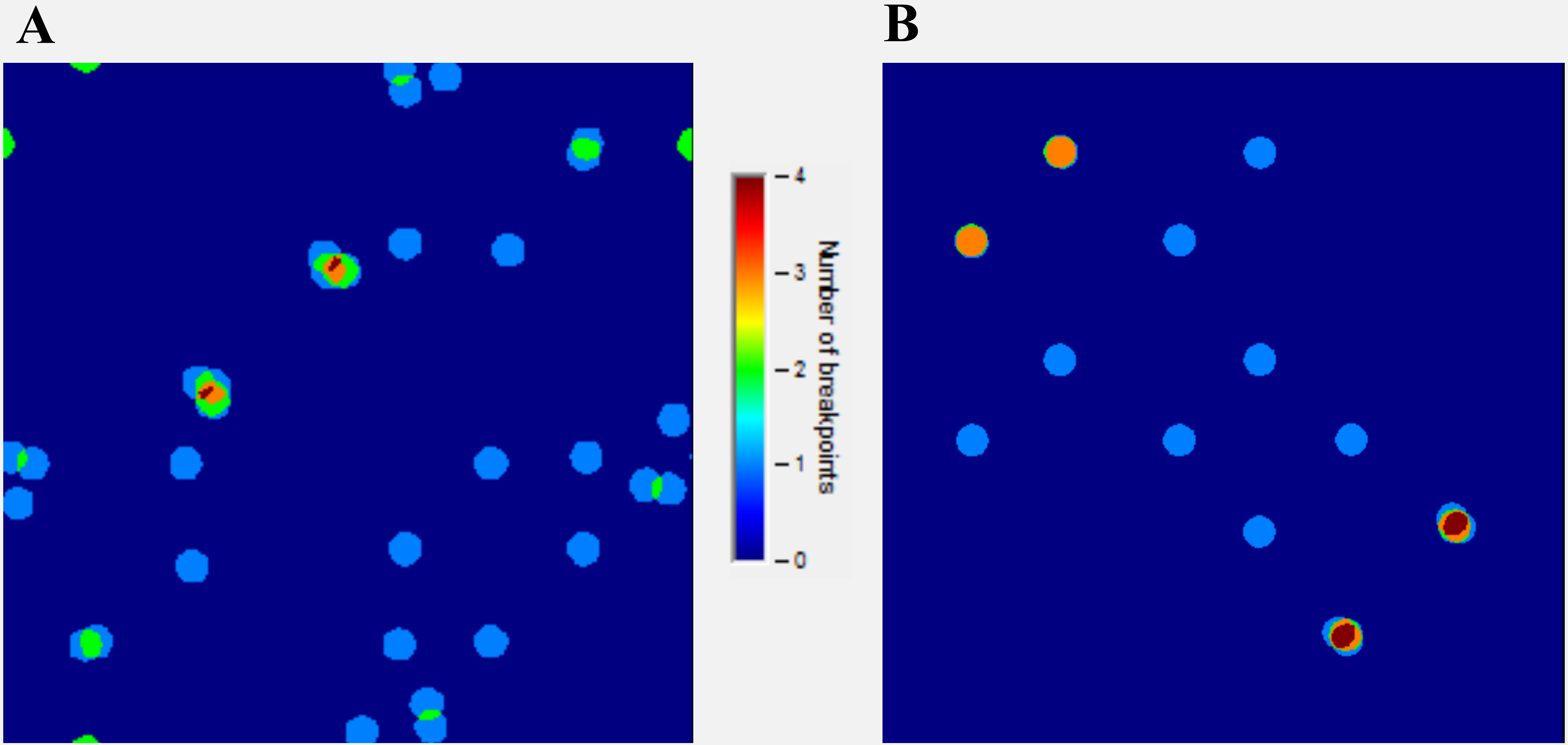

Table S4.

Event number	Recombinant (Recombinat Score)	Major parent (Recombinat Score)	Minor parent (Recombinat Score)	RDP	GENECONV	BootScan	MaxiChi	Chimaera	SiScan	3Seq	Observations
1#	ST358-VN/ND	ST225-VNI	ST267-VNIV								
2	ST365-VN/ND (0.590)	ST225-VNI (0.368)	ST267-VNIV (0.041)	9	9	10	10	5	6	10	Possible missidentification of recombinant<br>ST225-VN1 maybe actual recombinant<br>Possible missalingment artifact
3	ST266-VNIV (0.470)	ST42-VNII (0.244)	Unknown (0.286)	99	99	92	99	80	21	99	Recombinant signal maybe atributable to a process other than recombination<br>Only one parental sequence in triplet<br>Possible missidentification of recombinant<br>ST42-VNII maybe actual recombinant
4	ST222-VNII (0.757)	Unknown (0.155)	ST42-VNII (0.087)	2	2	-	2	2	1	2	Only one parental sequence in triplet
5	ST173-VNII (0.475)	ST258-VN/ND (0.156)	Unknown (0.369)	2	2	2	2	2	1	2	Only one parental sequence in triplet<br>Possible missidentification of recombinant<br>ST258-VN/ND maybe actual recombinant<br>ST225-VNI maybe actual recombinant
6	ST356-VN/ND (0.863)	ST219-VNI (0.000)	Unknown (0.137)	4	4	4	4	4	3	4	Only one parental sequence in triplet
7	ST126-VNIV (0.927)	ST181-VNI (0.068)	ST414-VNB (0.005)	3	7	7	7	7	3	7	
8	ST363-VN/ND (0.646)	ST253-VN/ND (0.256)	ST267-VNIV (0.098)	-	1	1	1	1	1	1	No ending breakpoint identified<br>Possible missidentification of recombinant<br>ST253-VN/ND maybe actual recombinant
9	ST343- VN/ND (0.774)	ST136-VNIV (0.000)	ST26-VNB (0.226)	-	4	4	6	5	-	6	Possible missidentification of recombinant<br>ST26-VN/ND maybe actual recombinant
10	ST260- VN/ND (0.788)	ST12-VNB (0.211)	ST276-VNIV (0.002)	3	2	-	3	-	1	1	Identify ending breakpoint position very uncertain<br>Possible missidentification of recombinant<br>ST12-VN/ND maybe actual recombinant
11	ST358- VN/ND (0.495)	ST103-VNI (0.377)	ST276-VNIV (0.128)	7	8	8	8	8	3	8	Possible missidentification of recombinant<br>ST103-VNI maybe actual recombinant
12	ST348- VN/ND (0.728)	ST267-VNIV (0.094)	ST26-VNB (0.178)	3	5	6	7	6	5	7	Possible missidentification of recombinant<br>ST126-VNB maybe actual recombinant
13	ST263- VN/ND (0.818)	Unknown (0.012)	ST47-VNI (0.170)	4	5	-	4	4	-	4	Only one parental sequence in triplet
14	ST249- VN/ND (0.596)	Unknown (0.286)	ST485-VNII (0.118)	5	1	1	5	1	1	4	Only one parental sequence in triplet<br>Possible missidentification of recombinant<br>ST26-VNB maybe actual recombinant
15	ST256- VN/ND (0.695)	ST126-VNIV (0.186)	ST232-VN/ND (0.120)	-	2	2	3	2	1	2	Recombinant signal maybe atributable to a process other than recombination<br>No ending breakpoint identified
16	ST253- VN/ND (0.680)	ST157-VNIV (0193)	ST232-VN/ND (0.127)	2	2	2	3	2	1	3	No ending breakpoint identified
17#	ST363- VN/ND	ST42-VNII	ST126-VNIV								
18	ST363- VN/ND (0616)	ST42-VNII (0.148)	ST154-VNIV (0.234)	-	1	-	1	1	1	1	Neither beginning nor ending breakpoints identified
19	ST478-VNB (0.429)	ST206-VNI (0.407)	ST12-VNB (0.164)	1	-	-	14	9	1	13	Possible missidentification of recombinant<br>ST206-VNI maybe actual recombinant<br>ST12-VNB maybe actual recombinant
20	ST220-VNII (0.627)	ST42-VNI (0.337)	ST396-VNB (0.037)	2	-	1	2	1	1	2	Recombinant signal maybe atributable to a process other than recombination<br>Possible missidentification of recombinant<br>ST42-VNII maybe actual recombinant
21	ST219-VNI (0.733)	ST414-VNB (0.199)	ST42-VNII (0.068)	-	-	-	7	5	-	5	Recombinant signal maybe atributable to a process other than recombination<br>Possible missidentification of beginning breakpoint
22	ST414-VNB (0.705)	ST12-VNB (0.289)	ST206-VNI (0.006)	-	-	-	9	-	-	1	Recombinant signal maybe atributable to a process other than recombination<br>Possible missidentification of ending breakpoint<br>Possible missidentification of recombinant<br>ST12-VNB maybe actual recombinant<br>ST206-VNI maybe actual recombinant
23	ST259_VN/ND (0.726)	ST103-VNI (0.153)	ST366-VN/ND (0.120)	-	4	3	1	-	-	5	Recombinant signal maybe atributable to a process other than recombination<br>Neither beginning nor ending breakpoints identified
24	ST30-VNI (0.801)	ST324-VN/ND (0.199)	Unknown (0.000)	5	-	8	40	-	-	37	Recombinant signal maybe atributable to a process other than recombination<br>Possible missidentification of ending breakpoint<br>Only one parental sequence in triplet
25	ST375-VN/ND (0.561)	Unknown (0.161)	ST231-VN/ND (0.278)	-	-	-	22	-	2	1	Possible missidentification of begining breakpoint<br>Only one parental sequence in triplet<br>Possible missidentification of recombinant<br>ST47-VNI maybe actual recombinant
26	ST482-VNB (0.390)	ST295-VNI (0.325)	Unknown (0.285)	-	-	-	102	1	3	-	Only one parental sequence in triplet<br>Possible missidentification of recombinant<br>ST295-VNI maybe actual recombinant<br>ST42-VNII maybe actual recombinant
27	ST362-VN/ND (0.584)	ST294-VNIV (0.316)	ST206-VNI (0.100)	-	1	1	1	1	-	1	Recombinant signal maybe atributable to a process other than recombination<br>begining breakpoint outside of confidence interval<br>No ending breakpoint idetntified<br>Possible missidentification of recombinant<br>ST294-VNIV maybe actual recombinant<br>ST206-VNI maybe actual recombinant

parameters were not calculated for RDP software

Table S5.

Event number	Recombinant (Recombinat Score)	Major parent (Recombinat Score)	Minor parent (Recombinat Score)	RDP	GENECONV	BootScan	MaxiChi	Chimaera	SiScan	3Seq	Observations
1	ST53-VGI (0.677)	ST318-VG (0.190)	ST360-VGII (0.134)	1	1	1	1	1	1	1	Recombination signal may be atribuitable to a process other than recombination
2	ST355-VGII (0.768)	ST316-VGII (0.135)	ST329-VGI (0.097)	1	2	2	2	2	2	2	
3	ST356-VG/ND (0.753)	ST329-VGI (0.247)	ST39-VGII (0.000)	1	2	2	2	2	2	2	Recombination signal may be atribuitable to a process other than recombination
4	ST357-VGII (0.762)	ST360-VGI (0.131)	ST356-VG/ND (0.107)	1	1	1	1	1	-	1	
5	ST181-VGII (0.736)	ST316-VGII (0.107)	ST166-VGIII (0.157)	2	2	2	3	3	3	3	Recombination signal may be atribuitable to a process other than recombination
6	ST71-VGII (0.694)	ST247-VGII (0.205)	ST64-VGIII (0.101)	-	1	1	1	1	1	1	Identified ending breakpoint position very uncertain
7	ST351-VG/ND (0.673)	ST319-VGI (0.274)	ST357-VGII (0.053)	-	1	1	1	1	1	1	Recombination signal may be atribuitable to a process other than recombination
8	ST351-VG/ND (0.703)	ST231-VGI (0.162)	ST246-VGII (0.135)	-	1	1	1	1	1	1	Recombination signal may be atribuitable to a process other than recombination
9	ST335-VGIII (0.719)	ST163-VGII (0.141)	ST231-VGI (0.140)	-	1	1	1	1	1	1	
10	ST223-VGIII (0.704)	ST326-VGI (0.173)	ST104-VGIV (0.123)	-	1	1	1	1	1	1	Recombination signal may be atribuitable to a process other than recombination
11	ST326-VGIII (0.000)	ST100-VGII*	Unknown*	-	1	1	1	1	1	1	Recombination signal may be atribuitable to a process other than recombination<br>Only parental sequence in triplet<br>Possible missidentification of recombinant<br>ST-100-VGIII may be actual recominant
12	ST71-VGII (0.000)	ST64-VGIII*	ST37-VGII*	-	-	1	1	1	-	1	Recombination signal may be atribuitable to a process other than recombination<br>No ending breakpoint identified

* Not calculable recombination scores
